# Increasing lipid yield in *Yarrowia lipolytica* through phosphoketolase and phosphotransacetylase expression in a phosphofructokinase deletion strain

**DOI:** 10.1186/s13068-021-01962-6

**Published:** 2021-05-04

**Authors:** Annapurna Kamineni, Andrew L. Consiglio, Kyle MacEwen, Shuyan Chen, Gamuchirai Chifamba, A. Joe Shaw, Vasiliki Tsakraklides

**Affiliations:** 1 Ginkgo Bioworks, 27 Drydock Ave, Boston, Massachusetts United States; 2 Belmont, Massachusetts United States

**Keywords:** Phosphotransacetylase, Phosphoketolase, Lipid yield, Cell-specific lipid productivity, *Yarrowia lipolytica*, Central carbon metabolism

## Abstract

**Background:**

Lipids are important precursors in the biofuel and oleochemical industries. *Yarrowia lipolytica* is among the most extensively studied oleaginous microorganisms and has been a focus of metabolic engineering to improve lipid production. Yield improvement, through rewiring of the central carbon metabolism of *Y. lipolytica* from glucose to the lipid precursor acetyl-CoA, is a key strategy for achieving commercial success in this organism.

**Results:**

Building on YB-392, a *Y. lipolytica* isolate known for stable non-hyphal growth and low citrate production with demonstrated potential for high lipid accumulation, we assembled a heterologous pathway that redirects carbon flux from glucose through the pentose phosphate pathway (PPP) to acetyl-CoA. We used phosphofructokinase (Pfk) deletion to block glycolysis and expressed two non-native enzymes, phosphoketolase (Xpk) and phosphotransacetylase (Pta), to convert PPP-produced xylulose-5-P to acetyl-CoA. Introduction of the pathway in a *pfk* deletion strain that is unable to grow and accumulate lipid from glucose in defined media ensured maximal redirection of carbon flux through Xpk/Pta. Expression of Xpk and Pta restored growth and lipid production from glucose. In 1-L bioreactors, the engineered strains recorded improved lipid yield and cell-specific productivity by up to 19 and 78%, respectively.

**Conclusions:**

Yields and cell-specific productivities are important bioprocess parameters for large-scale lipid fermentations. Improving these parameters by engineering the Xpk/Pta pathway is an important step towards developing *Y. lipolytica* as an industrially preferred microbial biocatalyst for lipid production.

**Supplementary Information:**

The online version contains supplementary material available at 10.1186/s13068-021-01962-6.

## Background

Lipids are important precursors in food, cosmetics, biodiesel and biochemical industries [1]. The ever-increasing demands of these industries are largely fulfilled by plant oil feedstocks [1, 2]. Plantations for oil are dependent on climatic changes, geopolitics and require large arable lands. Oil plantations are continually displacing large areas of tropical forests worldwide, severely affecting their regional biodiversity [2, 3]. These environmental and sustainability concerns have fueled research into microbial oil as an alternative source of lipid production [4, 5].

In recent years, *Yarrowia lipolytica* has emerged as the preferred organism to study and engineer lipid production [6]. It is an oleaginous yeast capable of accumulating more than 20% of its biomass as lipids [7] and with a few genetic modifications can accumulate over 60% lipid [8–10]. *Y. lipolytica*-derived products have received Generally Regarded As Safe (GRAS) status, and the yeast has a well-developed genetic tool kit for manipulating substrate and product pathways [6]. Lipids in *Y. lipolytica* are mostly stored as triacylglycerol (TAG) molecules (95%), composed mainly of C16 and C18 fatty acids with varying degrees of saturation [11]. Lipid composition alterations to make industrially relevant products like triolein have also been demonstrated in *Y. lipolytica* [12]. Consequently, *Y. lipolytica* is often chosen as a model organism to study the glucose-to-lipid pathway, lipid body biogenesis and homeostasis [4, 13, 14].

**Fig. 1:**
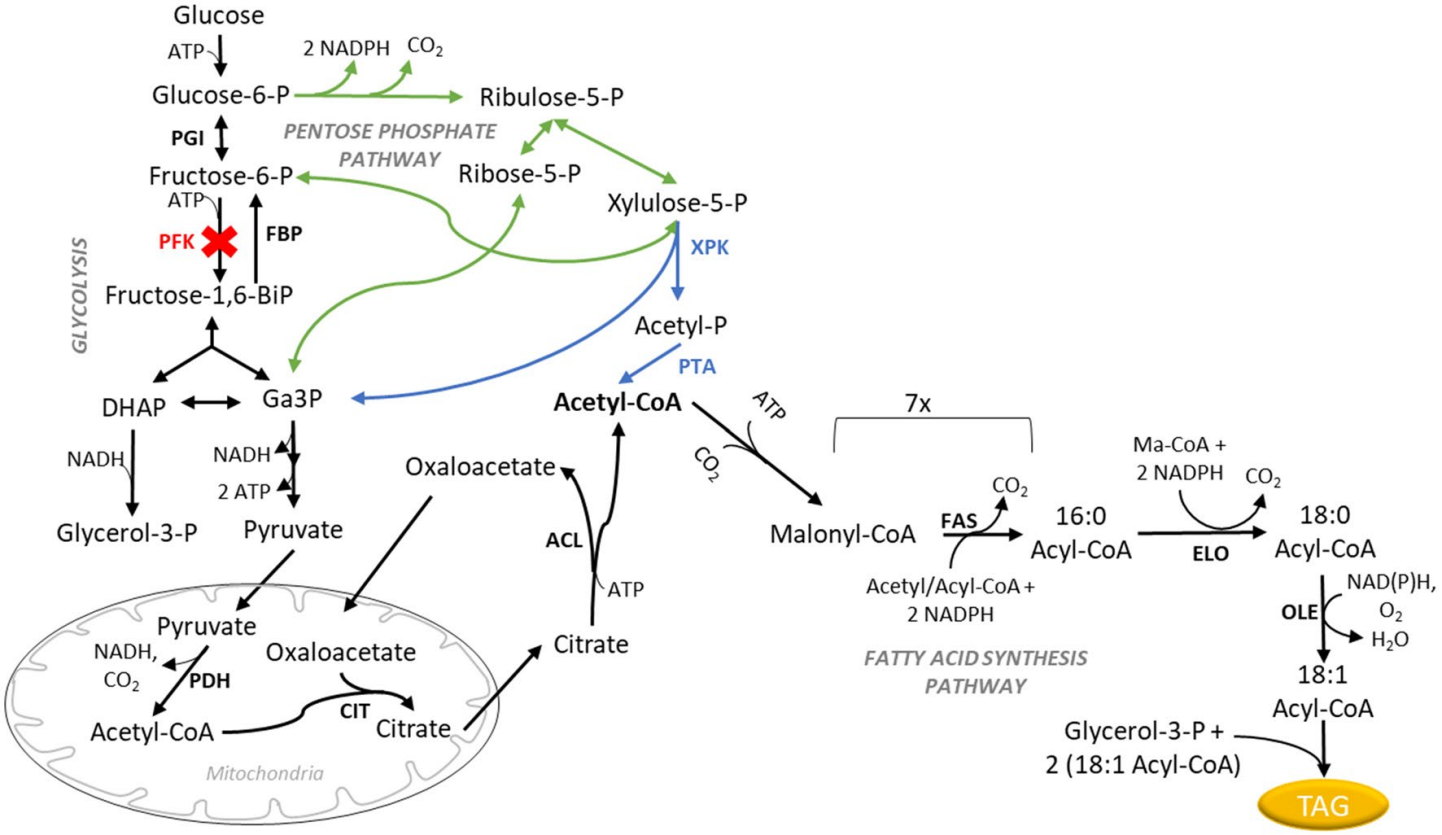
Lipid synthesis pathway for triolein production in *Y. lipolytica*. The native glucose to lipid synthesis pathway is shown in black, the pentose phosphate pathway is shown in green, the heterologous Xpk/Pta pathway is shown in blue and *PFK1* deletion in red. List of enzymes: *ACL* ATP:citrate lyase, *CIT* citrate synthase, *ELO* elongase, *FAS* fatty acid synthase, *FBP* fructose 1,6-bisphosphatase, OLE – Δ9 fatty acid desaturase, *PDH* pyruvate dehydrogenase, *PFK* phosphofructokinase, *PGI* phosphoglucoisomerase, *PTA* phosphotransacetylase, *XPK* phosphoketolase. *DHAP* dihydroxyacetone phosphate, *Ga3P* glyceraldehyde 3-phosphate, *Ma-CoA* malonyl-CoA

De novo fatty acid synthesis requires a constant supply of the metabolic precursor cytosolic acetyl-CoA and the reducing cofactor NADPH (nicotinamide adenine dinucleotide phosphate) [4, 13]. In *Y. lipolytica,* glucose is converted to cytosolic acetyl-CoA through the combined reactions of the glycolytic cycle, mitochondrial pyruvate dehydrogenase (PDH) and ATP:citrate lyase (ACL) (Fig. 1) [7]. The pentose phosphate pathway (PPP) has been shown to supply NADPH for lipid production [15]. The lipid synthesis pathway converts cytosolic acetyl-CoA to C16–18 fatty acyl-CoAs through a series of condensation reactions. Two molecules of NADPH are used for every acetyl unit condensed into a growing C16 fatty acyl chain. Elongating C16 fatty acyl-CoAs to C18 fatty acyl-CoAs also uses two NADPH molecules [16]. While fatty acid desaturation also requires reduced cofactors, it is unclear if the preferred cofactor is NADH or NADPH [16, 17]. Thus, to make one molecule of triolein (C_57_H_104_O_6_) using this biochemical pathway, 27 acetyl-CoA and at least 48 NADPH molecules are needed (Additional file 1: Fig. S1a). To produce the required intermediates, the native lipid pathway utilizes 18 glucose molecules (Additional file 1: Fig. S1a, b). We calculate a theoretical triolein yield from glucose at 0.27 g/g (Eq. 1).

1 $${\text{18 glucose}} \to {\text{27 Acetyl}} - {\text{CoA }} + {\text{ 48 NADPH}} \to {\text{1 C18}}:{\text{1 TAG}}$$

Metabolic engineering of the native pathway has improved lipid production in *Y. lipolytica* to nearly the theoretical maximal yield of 0.27 g lipid/g glucose [9, 18–22], when taking into account synthesis of non-lipid yeast biomass. Engineering strategies include overexpressing genes directly implicated in TAG synthesis [8, 19, 23, 24], rerouting glucose flux toward lipid precursor production [18] and deleting genes involved in lipid degradation [18, 25] and by-product formation [26]. Combined, these methods maximize flux and productivity through the native pathway to increase lipid production.

Another strategy to improve glucose-to-lipid production involves rewiring central carbon metabolism to improve yield of the biosynthetic lipid precursor acetyl-CoA, the redox cofactor NADPH, or both [22, 27–30]. This increases the theoretical maximum yield of the pathway and usually involves introducing heterologous enzymatic activities and creating new pathways in the organism [27]. One such pathway is the phosphoketolase (PK or Xpk)/phosphotransacetylase (Pta) pathway which has been tested in various organisms including *Escherichia coli* [31], *Saccharomyces cerevisiae* [32–35] and *Y. lipolytica* [28, 30, 36]. Phosphoketolase (Xpk, EC 4.1.2.9, EC 4.1.2.22) converts fructose 6-phosphate (F6P) and/or the PPP intermediate xylulose 5-phosphate (X5P) to acetyl phosphate (AcP) and glyceraldehyde 3-phosphate (Ga3P). Phosphotransacetylase (Pta, EC 2.3.1.8) catalyzes the reversible conversion of AcP to acetyl-CoA. The combined activities of Xpk and Pta produce cytosolic acetyl-CoA from the PPP instead of glycolysis, and thus link it to NADPH production. The Xpk/Pta route towards acetyl-CoA and NADPH is more efficient than *Y. lipolytica*’s native route via glycolysis and ACL [27] (Additional file 1: Fig. S1c). Overall, the Xpk/Pta pathway (Eq. 2) requires 2.3 fewer moles of glucose to make one mole of triolein compared to the native pathway. This increases the theoretical maximal yield of triolein to 0.31 g/g glucose compared to the native pathway (comparing Eqs. 1 and 2).

### Xpk/Pta pathway

2 $${15}.{\text{7 glucose}} \to {\text{27 Acetyl}} - {\text{CoA }} + {\text{ 48 NADPH}} \to {\text{1 C18}}:{\text{1 TAG}}$$

Work in *Y. lipolytica* has shown that expression of Xpk/Pta can lead to improved lipid phenotypes. Coexpression of *Aspergillus nidulans* phosphoketolase (An*XPK*) and *Bacillus subtilis* phosphotransacetylase (Bs*PTA*) led to improved yield from glucose [30] and increased lipid content from xylose [36]. Expression of *Leuconostoc mesenteroides* phosphoketolase (Lm*XPK*) and *Clostridium kluyveri* phosphotransacetylase (Ck*PTA*) led to higher dry cell weight and lipid content with a moderate increase in lipid yield [28]. Enzymatic activity was not measured, but improved lipid metrics suggested active Xpk/Pta pathways in the engineered strains. These studies were performed in Po1 series strains, derived from a set of backcrosses between the French and American *Y. lipolytica* wild-type strains W29 and CBS6142-2 [37, 38]. Although well characterized, the Po1 strains exhibit certain traits undesirable for commercial production: auxotrophy, significant citrate production and tendency to grow in the hyphal morphology under certain stresses [37, 39]. Wild-type *Y. lipolytica* strain YB-392 is a prototroph that has demonstrated potential for very high lipid content while growing entirely in the yeast morphology and with minimal citrate production [21]. It has been established that different *Y. lipolytica* genetic backgrounds respond differently to genetic engineering, even within the Po1 series [36]. Implementation of an Xpk/Pta pathway in YB-392 therefore remained to be investigated.

In published implementations of the Xpk/Pta pathway in other species, additional engineering was required for successful pathway utilization. In *E. coli*, 11 gene overexpressions, 9 gene deletions and over 50 genomic mutations accumulated through evolution were engineered to successfully rescue growth in a glycolytic mutant through the Xpk/Pta pathway [31]. In a farnesene-producing *S. cerevisiae* strain, the aldehyde dehydrogenase genes *ALD2* and *ALD6* were deleted to make Xpk/Pta the sole source of acetyl-CoA production. Additionally, the glycerol-3-phosphate phosphatase gene *RHR2* was deleted as this enzyme exhibited acetyl phosphatase activity and competed with *PTA* for the substrate AcP [35]. The removal of competing reactions ensures that Xpk/Pta is the only available route for growth and/or acetyl-CoA production.

In this study, we engineered the Xpk/Pta pathway in a glycolysis-deficient *pfk Y. lipolytica* strain in the YB-392 strain background. Phosphofructokinase (Pfk, EC 2.7.1.11) catalyzes the irreversible production of fructose 1,6-bisphosphate from fructose 6-phosphate (F6P). Carbon flux from glucose to lipids can still move through glycolysis in an otherwise unmodified Xpk/Pta strain, whereas deleting *PFK* is expected to reroute glucose flux through the PPP [40, 41] and in turn through the Xpk/Pta pathway (Fig. 1). Increased flux through the PPP could result in excess NADPH with negative consequences for growth [42, 43], unless sufficient NADPH-oxidizing reactions are present to restore the redox balance. We hypothesized that introduction of the Xpk/Pta pathway into a *pfk*-deficient *Y. lipolytica* strain would correct the NADPH imbalance by providing a route towards the NADPH-oxidizing lipid synthesis pathway (Fig. 1). The Xpk/Pta/*Δpfk1* strains engineered in the current study overcame the growth and lipid production deficits of the parent *Δpfk1* strain. They also exhibited improved lipid yield and cell-specific lipid productivity over the wild-type. Our work is the first in demonstrating phosphoketolase and phosphotransacetylase enzymatic activities as well as combination of the Xpk/Pta pathway with a *PFK* deletion in *Y. lipolytica*, maximizing the effect of the heterologous pathway towards lipid production. YB-392-derived *Y. lipolytica* strains using the Xpk/Pta/*Δpfk1* pathway would be well-suited for industrial applications as increased yield and cell-specific lipid productivity directly tie into better economics for lipid production.

## Results and discussion

In this study, we chose the wild-type *Y. lipolytica* strain YB-392 as the starting strain for its desirable biocatalyst qualities such as native lipid accumulation levels, minimal citrate formation, non-hyphal morphology and ease of genetic manipulation [10, 39]. This strain has also been used to study and engineer lipid pathway to improve lipid accumulation and alter lipid composition [10, 12].

### Identification of functional heterologous phosphotransacetylase (*PTA*) and phosphoketolase (*XPK*) in *Y. lipolytica*

**Fig. 2:**
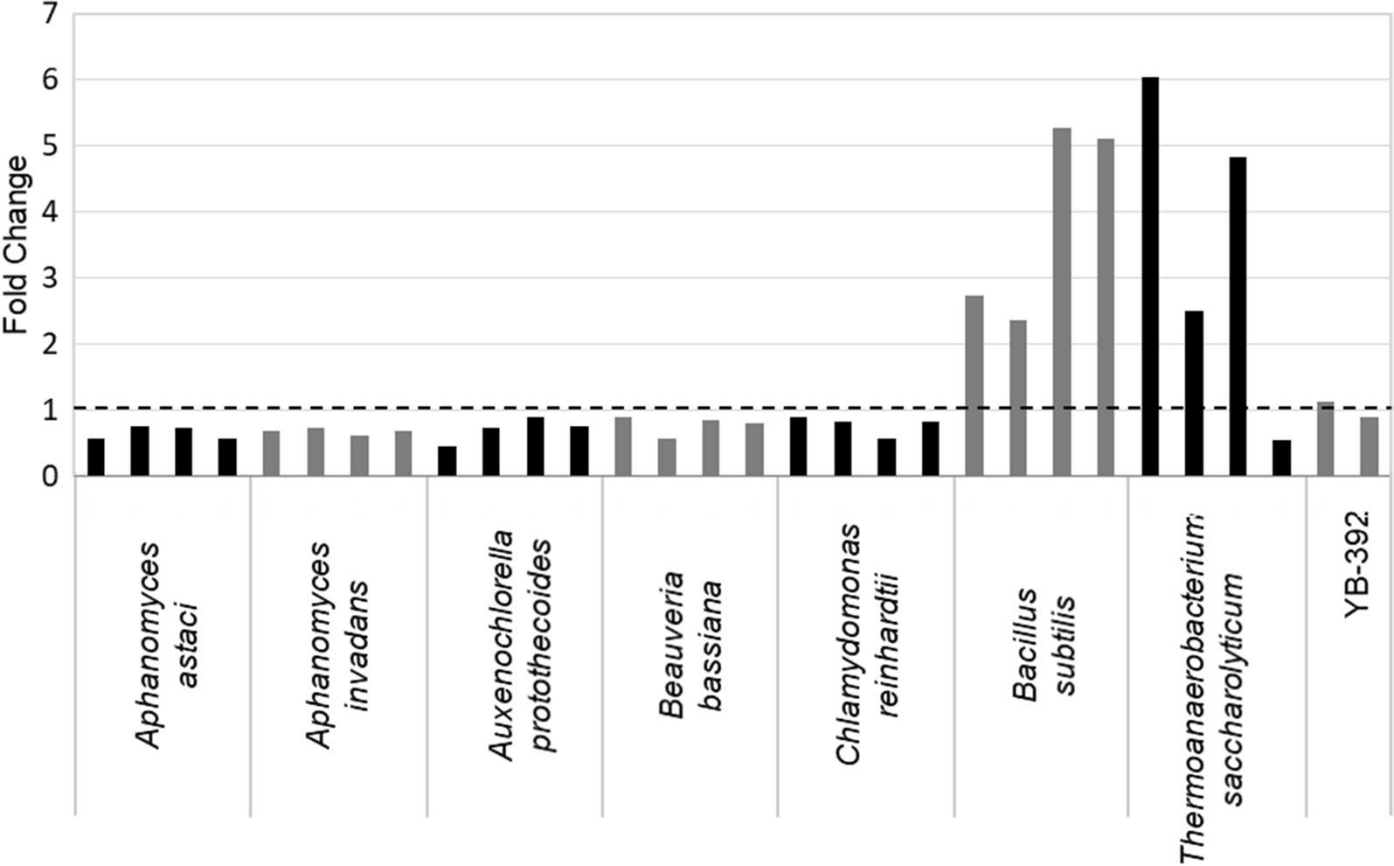
Heterologous Pta activity in *Y. lipolytica*. *PTA* genes from seven different source organisms codon-optimized to *S. cerevisiae* (GeneArt) were expressed in YB-392 under the control of the *Y.* *lipolytica* *EXP1* promoter using linear integrating expression cassettes. Cell-free extracts (CFE) from four transformants per test gene were analyzed for Pta activity using a DTNB assay. Data are presented as fold change of the measured specific activity over the averaged specific activity of the parent strain YB-392 (dashed line), which is included as control

**Fig. 3:**
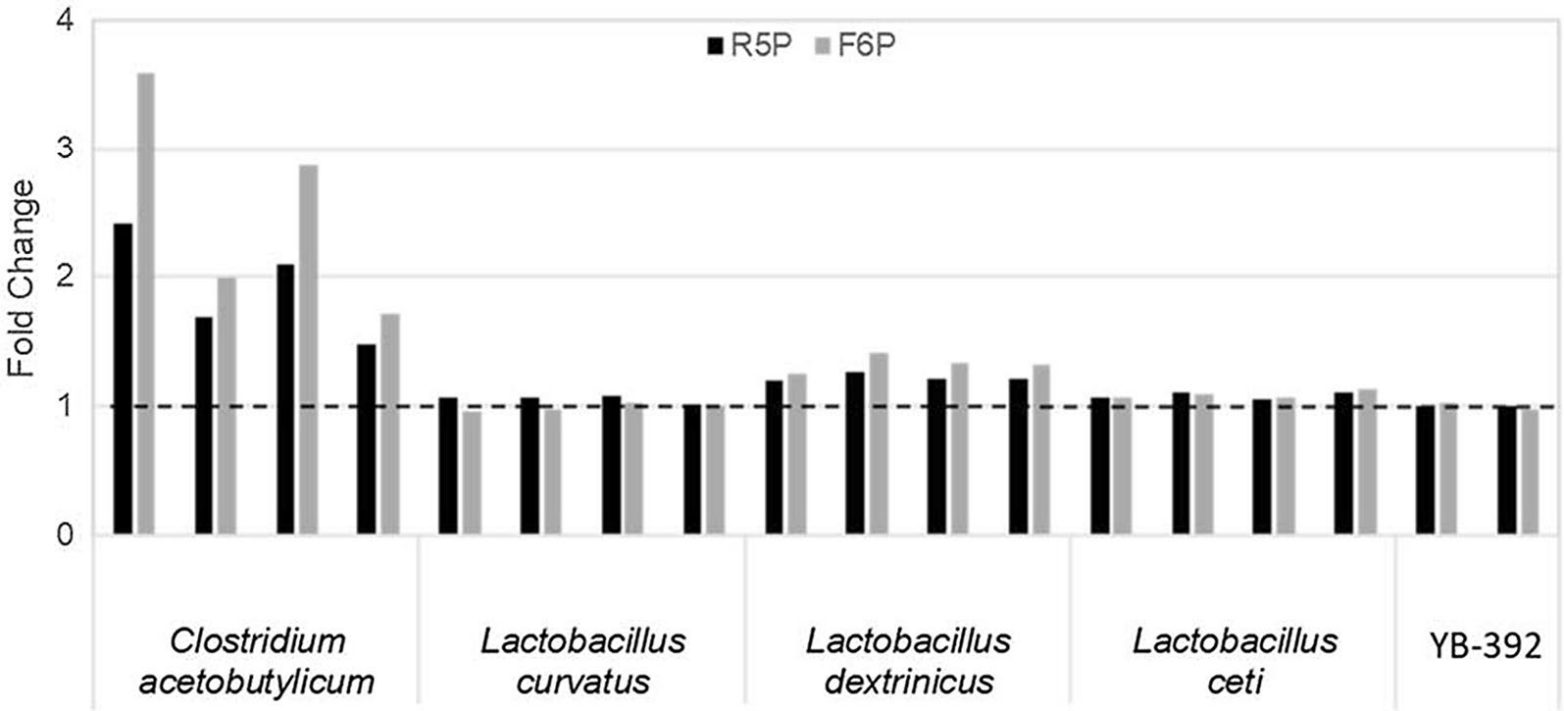
Heterologous Xpk activity in *Y. lipolytica*. *XPK* genes from four source organisms codon-optimized to *Y. lipolytica* (ATGme [59]) were expressed in YB-392 under the control of the *Y. lipolytica* *TEF1* promoter on replicating plasmids. Cell-free extracts from 4 transformants per test gene were analyzed by ferric hydroxamate assay to measure Xpk activity on ribose 5-phosphate (R5P, black bars) and fructose 6-phosphate (F6P, grey bars). Absorbance was measured at 540 nm and normalized to total protein in the crude cell-free extract after a reaction time of 30 min. Data are presented as fold change of the normalized absorbance over the averaged normalized absorbance of the parent strain YB-392 (dashed line), which is included as control

Heterologous *PTA* and *XPK* genes from various species of bacteria, archaea, algae and fungi were tested for activity in *Y. lipolytica*. Genes were individually expressed in wild-type strain YB-392 and cell-free extracts were assayed to measure Pta and Xpk activity (lists of all the *PTA* and *XPK* genes tested are in Additional file 1: Tables S1.1 and S1.2, respectively). Lm*XPK*, An*XPK*, Ck*PTA* and Bs*PTA* were previously expressed in the *Y. lipolytica* Po1 lineage [28, 30, 36]. Xpk and Pta activities were not reported in those studies and, Lm*XPK* and An*XPK* did not exhibit activity in our wild-type strain YB-392 (Additional file 1: Table S1.2). The wild-type strain contains no endogenous Xpk or Pta and shows background levels of activity. Strains expressing *PTA* genes from *Bacillus subtilis* (Bs*PTA*(v1)) and *Thermoanaerobacterium saccharolyticum* and an *XPK* gene from *Clostridium acetobutylicum* (Ca*XPK*(v1)) exhibited the highest activity in our screens (Figs. 2 and 3). This marks the first published measurement of Pta and Xpk activity in *Y. lipolytica*.

In the course of identifying active genes in *Y. lipolytica*, we found that different methods of screening were successful for *PTA* and *XPK*. Linear integrating expression cassettes and codon optimization to *S. cerevisiae* resulted in successful *PTA* expression. This approach did not yield detectable Xpk activity in our study (data not shown) presumably due to low expression from the candidate genes. Codon optimization of *XPK* genes to *Y. lipolytica* and expression from replicating plasmids, a method of expression that yields uniform high expression, was required to identify one gene candidate with detectable enzymatic activity (Fig. 3).

### Construction and characterization of the *Y. lipolytica Δpfk1* strain (NS1047)

To increase glucose flux through the Xpk/Pta pathway, we partially disabled glycolysis by deleting the *PFK1* gene (Fig. 1). *Y. lipolytica* contains only one *PFK* gene (*YALI0D16357*) and its deletion leads to loss of growth on minimal media with glucose as the only carbon source [44]. We deleted the *PFK1* gene in YB-392 to create the *Δpfk1* strain NS1047.

**Fig. 4:**
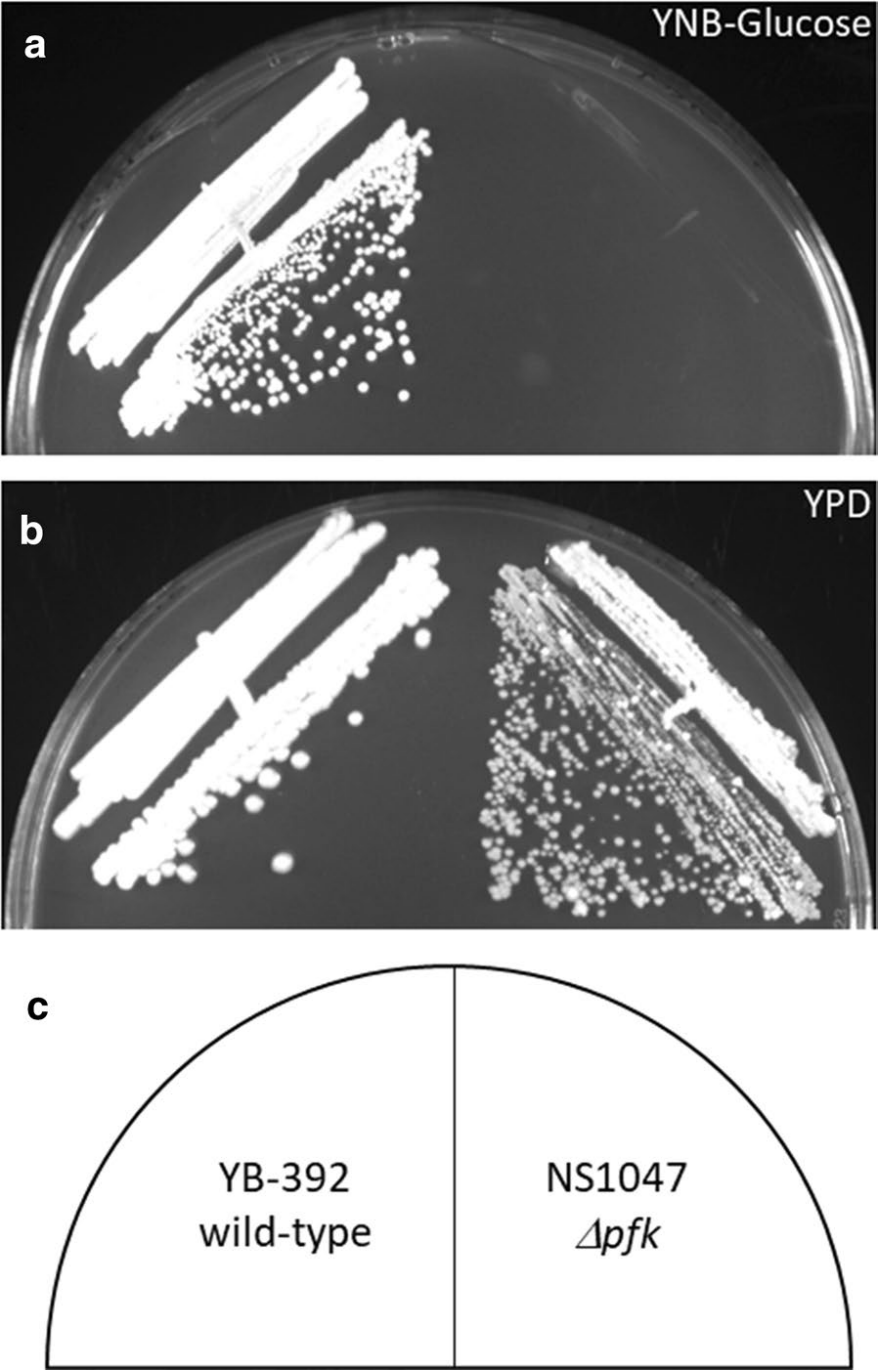
*Y. lipolytica* growth on glucose. Strains YB-392 (wild-type) and NS1047 (*Δpfk1*) were streaked on A) Yeast Nitrogen Base (YNB) media containing 2% glucose for 4 days, or B) YPD for 2 days as labeled in C

**Fig. 5:**
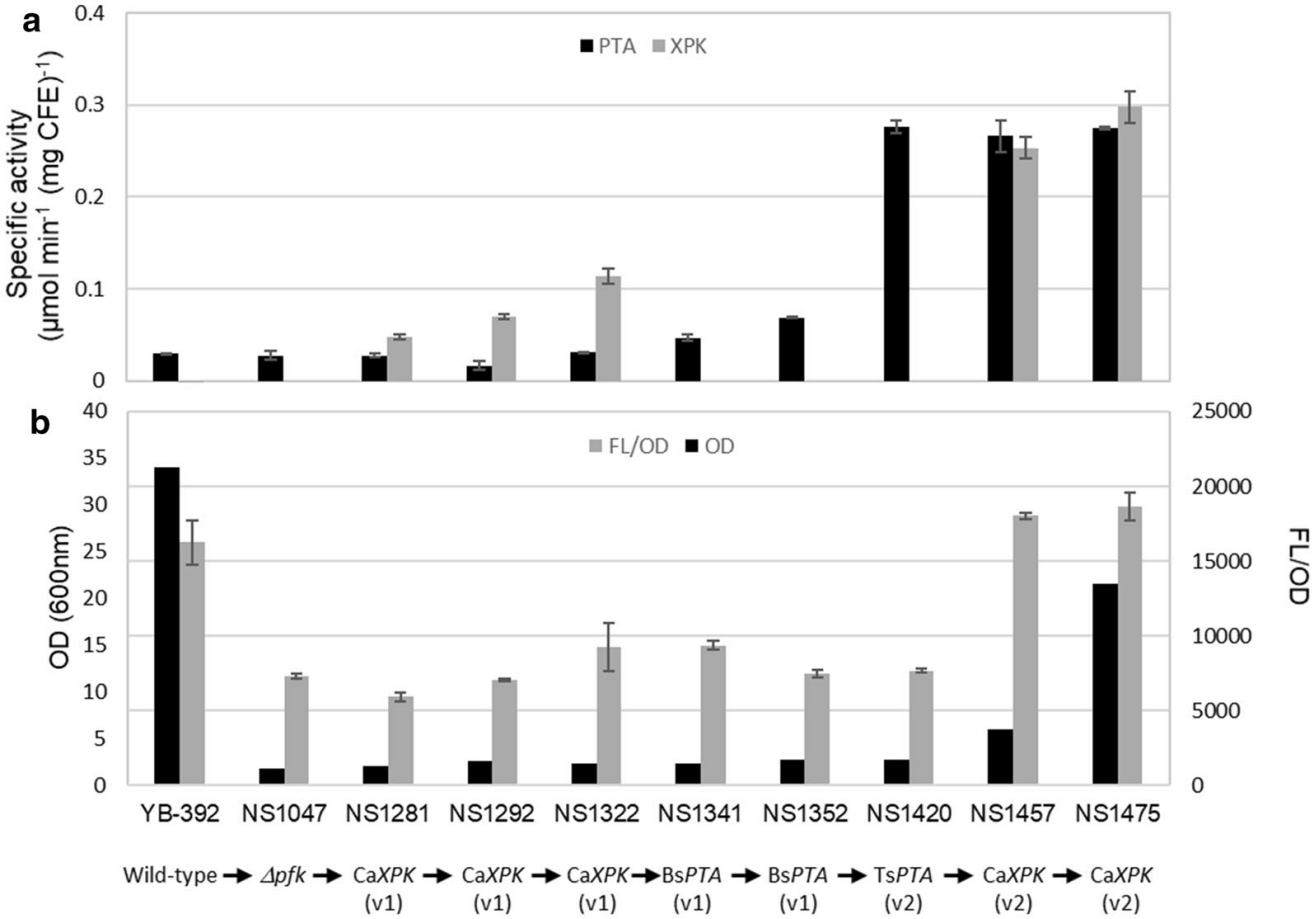
Engineering the Xpk/Pta pathway in a *Δpfk1 Y. lipolytica* strain. **a** Pta and Xpk activity. Pta activity in all strains shown was measured using the DTNB assay (black bars). Xpk activity in the control strain YB-392 and all the strains that obtained a copy of Ca*XPK* through transformation (NS1281, NS1292, NS1322, NS1457 and NS1475) was measured using the ferric hydroxamate assay with ribose 5-phosphate as the substrate (grey bars). NS1047, NS1341, NS1352 and NS1420 were excluded from this assay. **b** Growth and lipid accumulation assays. OD_600_ was measured after 2 days of growth in lipid production media (black bars). Lipid accumulation was measured as fluorescence/OD after overnight growth in glycerol followed by seven days of culture in modified Verduyn media (grey bars). Modified Verduyn media contained glucose as the only carbon source and no nitrogen to induce lipid production

Disruption of glycolysis through *PFK* or *PGI* deletions can cause growth defects on glucose [40, 42], presumably due to excess NADPH produced by increased glucose flux through the PPP [43, 45]. In organisms that can metabolize cytosolic NADPH, glycolytic-deficient strains retain or regain growth on glucose (e.g., through native cytosolic NADPH oxidase in *Kluyveromyces lactis* [43] or by overexpression of a transhydrogenase enzyme in *E. coli* [45]). *Y. lipolytica* lacks cytosolic NADPH oxidase [46] and homologues to *PFK1* [44]. Furthermore, NS1047 was unable to grow in minimal glucose media (Fig. 4a) and lipid production media (Fig. 5b, black bar) with glucose as the primary carbon source. We therefore hypothesize that *Y. lipolytica* cannot tolerate the excess NADPH produced when glucose is consumed in a *PFK*-deleted strain. Growth on YPD was not abolished, but it was slower for NS1047 than YB-392 (Fig. 4b). The reduced severity of the growth defect on this rich medium could be attributed to the ability of *Y. lipolytica* to utilize amino acids for growth [47] and the ability of a *pfk1* deletion mutant to metabolize permissive carbon sources in the presence of glucose [44].

### Engineering the Xpk/Pta pathway into a *Δpfk1* strain

To assemble the Xpk/Pta/*Δpfk1* pathway in *Y. lipolytica*, we expressed Ca*XPK*(v1) and Bs*PTA*(v1) in NS1047 (*Δpfk1)* (Fig. 5). With each transformation step, we screened for the best transformant using appropriate enzymatic assays and measured growth and lipid accumulation on glucose (Additional file 1: Table S2.1). Noticeable Xpk and Pta activities were obtained in the course of constructing strain NS1352 (Fig. 5a). However, NS1352 containing three copies of Ca*XPK*(v1) and two copies of Bs*PTA*(v1), showed minimal improvement in growth or lipid accumulation on glucose when compared to NS1047 (Fig. 5b). One possibility for the lack of improvement is that Xpk and Pta activities were still too low for a functional pathway. As addition of multiple copies of Ca*XPK*(v1) and Bs*PTA*(v1) genes yielded only incremental improvements in enzymatic activities (Fig. 5a), we decided to revisit codon optimization strategies to improve gene expression.

To further improve Xpk and Pta activity in *Y. lipolytica*, different codon optimization strategies were tested on our top performing genes Bs*PTA*, Ts*PTA* and Ca*XPK*. Ts*PTA* codon-optimized to *Y. lipolytica* (GeneArt) exhibited the highest activity and is referred to as Ts*PTA*(v2) from hereon. Improving Xpk activity continued to be challenging and no improvements were observed using the strategies that were successful with Pta (data not shown). Through analysis of publicly available *Y. lipolytica* transcriptomics data (data accessible at NCBI GEO database [48–50]), we noted that the highest expressed genes contained few rare codons (Additional file 1: Fig. S2a). Based on this observation, we manually designed and tested three additional versions of Ca*XPK* (Additional file 1: Fig. S2b). The highest Xpk activity was obtained when all codons present at a frequency ≤ 2% were replaced with their higher frequency counterparts and this gene is referred to as Ca*XPK*(v2).

To determine whether Ts*PTA*(v2) and Ca*XPK*(v2) increase flux through the Xpk/Pta pathway, we tested whether their expression in NS1352 could restore growth and lipid accumulation on glucose. Addition of Ts*PTA*(v2) quadrupled Pta activity (NS1420, Fig. 5a), but growth and lipid production on glucose remained unchanged (Fig. 5b), suggesting that Xpk activity could still be limiting. Consistent with this hypothesis, addition of Ca*XPK*(v2) improved lipid accumulation to near YB-392 levels (NS1457, Fig. 5b, grey bars). Growth on glucose also improved, but was still deficient compared to YB-392 (NS1457, Fig. 5b, black bars). Addition of a second copy of Ca*XPK*(v2) almost quadrupled growth on glucose compared to its parent strain (NS1457 and NS1475, Fig. 5b, black bars). Lipid accumulation remained similar to the parent strain NS1457 and YB-392 (Fig. 5b, grey bars). The observation that lipid accumulation was restored with fewer copies of *XPK* than were required to restore wild-type growth to a *Δpfk1* strain suggests that more Xpk activity is needed for growth than lipid production.

**Fig. 6:**
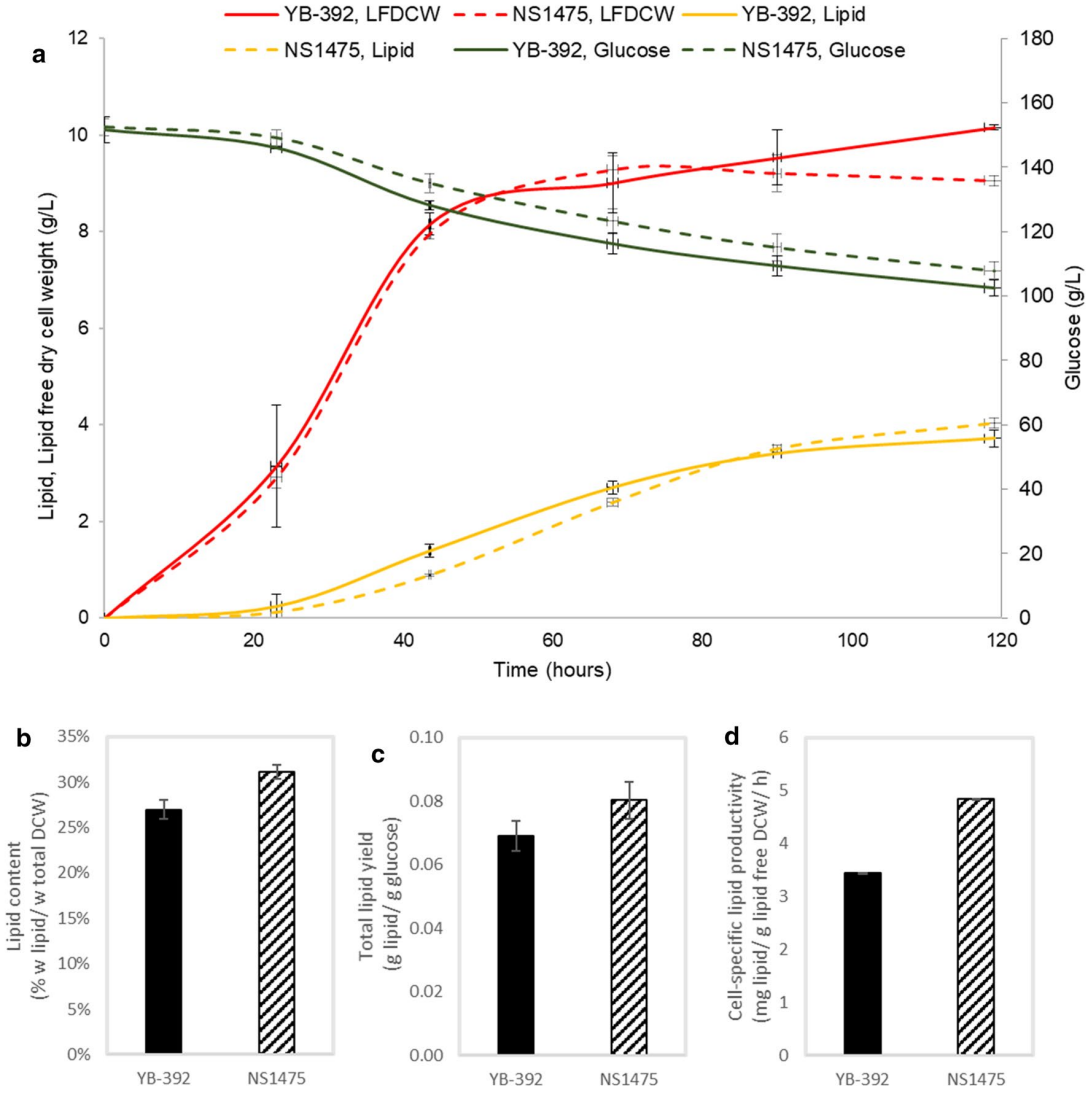
Characterization of Xpk/Pta/*Δpfk1* strain NS1475 and YB-392 in 1-L glucose batch fermentation. **a** Time-course profiles of glucose consumption, lipid free dry cell weight (LFDCW) and lipid titers of YB-392 and NS1475 over a 5-day fermentation. **b** Lipid content. **c** Total lipid yield. **d** Cell-specific lipid productivity (day 2 – day 5). Data are mean ± standard deviation for two replicate runs

To further characterize the Xpk/Pta/*Δpfk1* pathway strain NS1475, we carried out batch fermentations in 1-L bioreactors with YB-392 included as control. Two replicate fermentation experiments comparing YB-392 and NS1475 were conducted on two separate occasions to account for any culturing variations. NS1475 was comparable to the wild-type YB-392 in terms of growth and total lipid accumulated (Fig. 6a). NS1475 recorded an improved total lipid yield (+ 16%), cell-specific lipid productivity (+ 41%) and lipid content (+ 16%) over YB-392 (Fig. 6b–d). Our results confirm that the Xpk/Pta pathway can rescue growth and lipid defects of a *PFK* deletion while also improving bioprocess parameters like yield and cell-specific productivity.

### Confirming improved lipid production with Xpk/Pta/*Δpfk1* genotype

**Fig. 7:**
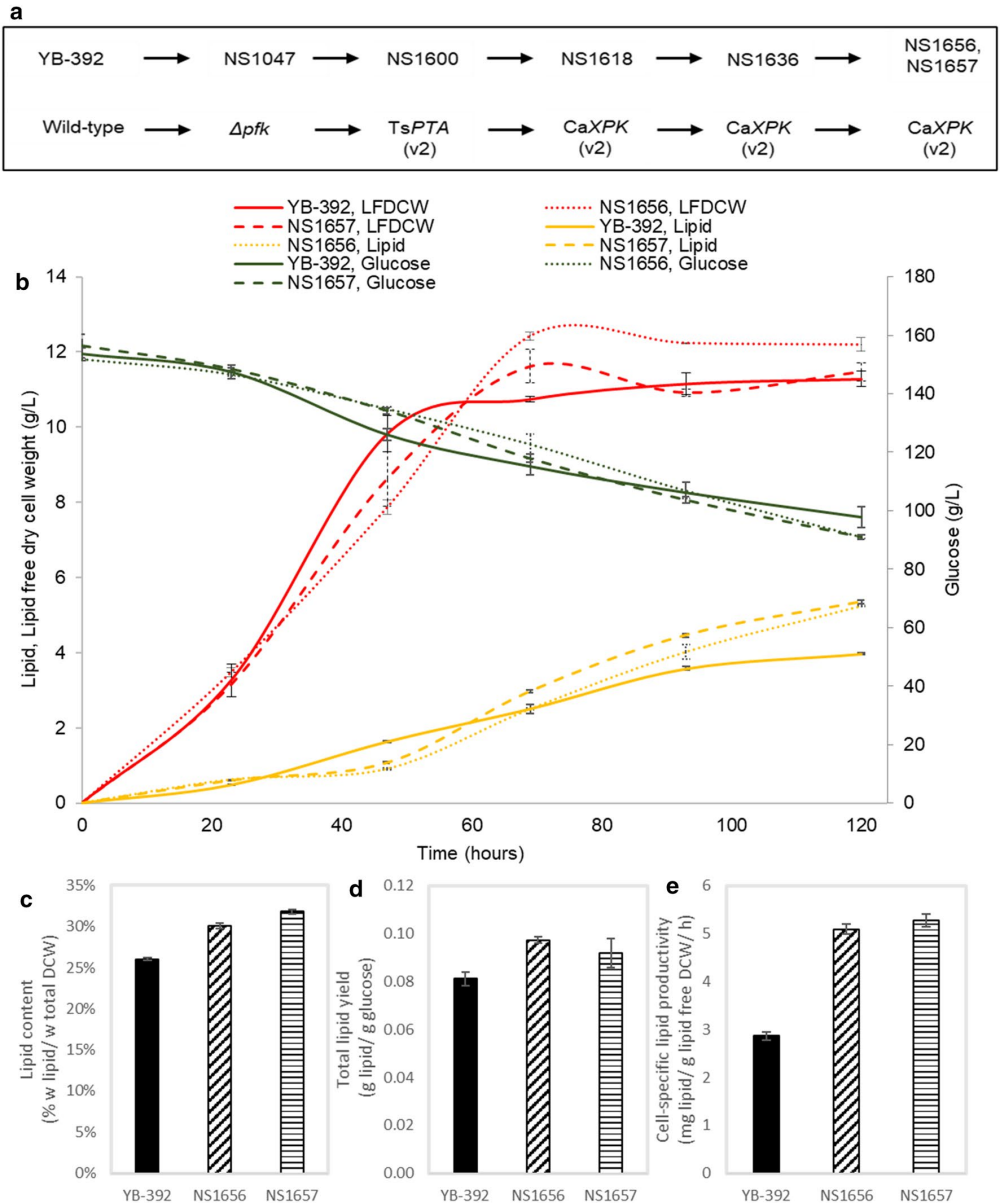
Rebuilding Xpk/Pta/*Δpfk1* in *Y. lipolytica* with Ts*PTA*(v2) and Ca*XPK*(v2). **a** Strain construction flowchart. **b** Time-course profiles of glucose consumption, lipid free dry cell weight (LFDCW), lipid titers of Xpk/Pta/*Δpfk1* strains NS1656-57 and YB-392 in 1-L glucose batch fermentations over a 5-day period. **C** Total lipid yield. **d** Cell-specific lipid productivity (day 2–day 5). **e** Lipid content. Data are mean ± standard deviation for two replicate runs

To confirm that the Xpk/Pta pathway was directly responsible for restoring growth and improving lipid production from glucose in *Δpfk1*, we reconstructed the pathway in NS1047 using only Ts*PTA*(v2) and Ca*XPK*(v2) (Fig. 7a). We wanted to eliminate the possibility that the nine rounds of transformation involved in constructing NS1475 resulted in unintentional contributions to the strain’s phenotype. As before, Pta and Xpk activity, growth on glucose, and lipid production were monitored at each engineering step (Additional file 1: Table S2.2). One copy of Ts*PTA*(v2) and three copies of Ca*XPK*(v2) were required to restore growth and lipid accumulation to YB-392 levels reducing the number of engineering steps to five instead of nine. Despite the usage of a more active *XPK* gene sequence, the pathway was again Xpk-limited and required more copies of *XPK* than *PTA* to achieve the desired phenotype. Xpk limitation was also reported by Lin et al*.* while engineering the pathway in *E. coli* [31]. An Xpk bottleneck could be increasingly restricting as we engineer strains toward higher lipid content by combining this pathway with native lipid pathway engineering (e.g., overexpression of *DGA1*). Additional research could identify a higher-expressing *XPK* gene that may further reduce the number of engineering steps required to introduce a functional Xpk/Pta/*Δpfk1* pathway in *Y. lipolytica.*

The two best-performing strains at the end of this engineering strategy (NS1656 and NS1657, Fig. 7a) were further characterized in 1-L batch fermentations alongside YB-392. Under the same fermentation conditions, all three strains attained similar lipid-free biomass (Fig. 7b). As was the case with NS1475, the Xpk/Pta/*Δpfk1* strains outperformed YB-392 in lipid yield and cell-specific lipid productivity (Fig. 7c and d). A slight improvement in lipid content was also observed (Fig. 7e). Overall, all three engineered strains showed an improvement in total lipid yield ranging from 13.3%—19.6% and an improvement in cell-specific productivity ranging from 41%—78% over YB-392.

These results confirm that we successfully rewired central carbon metabolism in *Y. lipolytica*. As expected, redox imbalance created by the *PFK* deletion led to growth and lipid accumulation defects on glucose media. *XPK/PTA* expression resulted in confirmed Xpk and Pta activity and corrected this imbalance by providing a route towards the NADPH-oxidizing lipid synthesis pathway, restoring growth and lipid production on glucose. Unlike *S. cerevisiae* (Meadows et al., 2016), we found that *Y. lipolytica* did not have any native competing AcP-consuming reactions (no AcP degradation detected in wild-type strain, data not shown). Thus, in engineering the Xpk/Pta pathway in *Y. lipolytica* we found that Xpk limitation was the main bottleneck and *PFK* deletion offers a means to ensure maximal pathway utilization.

## Conclusions

To achieve maximum lipid titer and yield from *Y. lipolytica*, a combination of native pathway engineering and rewiring of central carbon metabolism may prove to be a successful strategy. In this study, we expressed optimized phosphoketolase and phosphotransacetylase genes in a phosphofructokinase-deficient Δ*pfk1* strain to demonstrate the use of Xpk/Pta pathway in improving lipid production in the commercially attractive YB-392 strain background. The engineered strains recorded up to 19% higher total lipid yield and up to 78% higher cell-specific productivity compared to the wild-type strain. Such improvements in bioprocess metrics make lipid production in *Y. lipolytica* more suitable for industrial applications. Since the Xpk/Pta pathway essentially improves acetyl-CoA production, this pathway can be used to improve bioprocess metrics of other acetyl-CoA derived products including fatty alcohols, sterols, alkenes/alkanes, isoprenoids, etc. [48]. The theoretical improvement in yield makes the Xpk/Pta pathway a compelling technology for large-scale, commodity fermentation in the biofuel and biochemical industries.

## Methods

### Strains, cultivation and media

Wild-type, haploid *Yarrowia lipolytica* strain YB-392 was obtained from the ARS Culture Collection (NRRL). For routine growth and genetic transformation, strains were cultured in YPD (10 g/L yeast extract, 20 g/L bacto peptone, 20 g/L glucose), YPD/Et/Gly (YPD as described, plus 20 g/L ethanol and 30 g/L glycerol) and YPG (10 g/L yeast extract, 20 g/L bacto peptone, 20 g/L glycerol) media at 30 °C. 20 g/L agar was added to prepare solid media. Selection was performed using 300 μg/mL hygromycin B (Corning), 500 μg/mL nourseothricin (Werner Bioreagents) or 30 μM 5-fluoro-2′-deoxyuridine (FUDR) (Fisher Scientific).

### *PFK1* deletion

The *Y. lipolytica PFK1* gene *YALI0D16357* was deleted through targeted genomic integration using direct repeats and a combination of positive and negative selection for marker recycling. Using standard molecular biology techniques, a construct was designed comprising the genetic parts listed in Additional file 1: Table S3. A two-fragment deletion cassette was amplified by PCR using a combination of terminal and internal oligonucleotide primers such that the fragments overlapped in the *nat* marker reading frame, but neither fragment alone contained the entire functional nourseothricin-resistance gene. PCR products were transformed into hydroxyurea-treated cells as described in our previous work [51]. Transformation recovery was in YPD/Et/Gly to provide carbon sources in addition to glucose. Transformed cells were plated on YPD/Et/Gly containing 500 µg/mL nourseothricin. Successful cassette integration replaced the *PFK1* locus by a double recombination event at the 47-bp upstream and 621-bp downstream regions. A longer downstream homology region was chosen to increase the likelihood of this recombination event as opposed to recombination between the homologous 450-bp regions in the integration cassette and upstream of *PFK1*. Nourseothricin-resistant colonies were screened by PCR for the presence of the expected targeted integration product and the absence of the *PFK1* gene. The phenotype of resulting deletion strains was confirmed by plating on defined media with glucose as the only carbon source.

To eliminate the marker cassette, the deletion strains were grown on YPD/Et/Gly agar plates without selection for 1 day to allow for survival of cells that naturally excised the cassette by recombination of the 450-bp direct repeat formed between the endogenous *PFK1* upstream region and the identical sequence introduced in the integration cassette. Subsequent plating of strains on YPD/Et/Gly agar containing 30 µM 5-fluoro-2′-deoxyuridine (FUDR) selected for the absence of the thymidine kinase gene. To identify marker-less *PFK1* deletion strains, FUDR-resistant isolates were screened for reversion to nourseothricin sensitivity and loss of the marker cassette from the *pfk1* locus was confirmed by PCR.

Oligonucleotide primer sequences are included in the Additional file 1: Table S3.1.

### *XPK* and *PTA* gene expression

To identify functional *XPK* and *PTA* genes, expression cassettes were transformed into the desired *Y. lipolytica* strains as a part of a linear integrated expression construct [10] or replicating plasmid composed of the genetic parts listed in Additional file 1: Tables S4.1 and S4.2. For replicating plasmids, 100 ng of undigested plasmid was used in the transformation mix. To assemble the Xpk/Pta/*Δpfk1* pathway, NS1047 and subsequent intermediate strains were transformed with linear constructs containing *XPK* or *PTA* and positive and negative marker expression cassettes (Additional file 1: Table S4.3). Transformants were selected on antibiotic plates and screened for the highest performance using appropriate enzymatic, lipid and growth assays. Additional file 1: Tables S2.1 and S2.2 describe the screening steps used to construct NS1475 and NS1656-57, respectively. To eliminate the marker cassette in these strains, the chosen isolates were grown on YPD agar plates without selection for one day to allow for survival of cells that naturally excised the cassette by recombination between the identical copies of the *Y. lipolytica TEF1* promoter driving expression of thymidine kinase and the gene of interest in the integration cassette. Subsequent plating on YPD agar containing 30 µM 5-fluoro-2′-deoxyuridine (FUDR) counter-selects for the thymidine kinase gene. FUDR-resistant isolates were screened by confirmation of reversion to nourseothricin sensitivity to identify marker-less strains.

### Growth and lipid assays

To evaluate growth on glucose, strains were patched on YNB plates (6.7 g/L Yeast Nitrogen Base without amino acids, 20 g/L agar) or cultured in lipid production media (0.5 g/L urea, 1.5 g/L yeast extract, 0.85 g/L casamino acids, 1.7 g/L Yeast Nitrogen Base without amino acids and ammonium sulfate, 100 g/L glucose, and 5.11 g/L potassium hydrogen phthalate) [10]. Growth in lipid production media was tested by growing strains overnight in YPD, washing with sterile water, and inoculating into the lipid production media at a starting OD_600_ (optical density measured at 600 nm) of 0.05. OD_600_ measurements to monitor growth were taken after culturing for 2 days in shake flasks.

To measure lipid accumulation, strains were grown in glycerol (YPG) for 24 h and then switched to a modified Verduyn media [52] (modified to contain no ammonium sulfate and 100 g/L glucose) to induce lipid production. The cells grown in YPG were pelleted, washed with water and resuspended in the modified Verduyn media and cultured for 7 days. These characterizations were carried out in 96-well, 48-well or 24-well deep well plates and 250-mL shake flasks. The lipid Bodipy assay described in our previous work [10] was used with one modification: PBS was used instead of the master mix previously described. Lipid accumulation was measured as fluorescence units normalized to the OD_600_ (Fl/OD).

### Cell-free extract preparation and enzymatic assays

Strains were grown in 5 mL YPD or YPG overnight at 30 ºC. The cells were pelleted by centrifugation and after a wash with autoclaved water, were pelleted again. The pellets were resuspended in lysis buffer Y-PER™ plus (Thermo Scientific) per the manufacturer’s instructions. Protease inhibitor cocktail (Sigma Aldrich) was added (5 µL for every 1 mL of the lysis buffer used) and 0.5-mm glass beads were added at an equal volume to the cell pellet. The cells were homogenized in a FastPrep-24™ 5G (MP biomedicals) (3 cycles of 5.5 m/s for 30 s, with 5 min resting on ice in between runs). The homogenized cell lysates were centrifuged at 10,000 rpm for 10 min at 4 ºC and the supernatants were stored on ice for immediate use in enzymatic assays. Total protein concentrations were determined by the Pierce™ Coomassie (Bradford) Protein Assay Kit (Thermo Scientific).

Phosphotransacetylase activity was quantified using Ellman’s thiol reagent, 5,5′-dithiobis-(2-nitrobenzoic acid) (DTNB) which reacts with Coenzyme A to form a mercaptide ion measurable at 412 nm [53], with an extinction coefficient of 13.5 mM^−1^ cm^−1^. The 1-mL reaction mixture contained 100 mM Tris–HCl (pH 7.2), 5 mM MgCl_2_, 5 mM KH_2_PO_4_, 0.1 mM DTNB, and 0.1 mM acetyl-CoA [54]. 10–40 µL of cell-free extract was mixed with the assay ingredients, with acetyl-CoA added at the end to start the reaction. Specific activity measurements were calculated.

Phosphoketolase activity was measured using a ferric hydroxamate assay on crude cell-free extracts [55]. The 200 µL reaction mixture contained 0.5 mM thiamine pyrophosphate (TPP), 1 mM DTT, 5 mM MgCl_2_, 50 mM morpholine ethane sulfonic acid (MES) buffer (pH 5.5 for all kinetic studies), 333 mM sodium phosphate substrate and 333 mM of either fructose 6-phosphate or ribose 5-phosphate as substrate. Ribose 5-phosphate which is converted to X5P by endogenous enzymes in cell-free extract, was used to measure phosphoketolase activity indirectly [56, 57]. 20–80 µL of cell-free extract was used to initiate the reaction, and the mixture was incubated at 37 ºC for 15–30 min. 100 µL of 2 M hydroxylamine hydrochloride (pH 7.0) was added and incubated at room temperature for 10 min to stop the reaction. 600 µL of a 1:1 mixture of 2.5% FeCl_3_ in 2 N HCl and 10% trichloroacetic acid was added. The final reaction step results in the formation of the ferric-hydroxamate complex, which was measured spectrophotometrically at 540 nm [58]. For specific activity measurements, reactions were stopped at 5-min intervals and ΔAbs/min was calculated.

### Codon optimizations

Bs*PTA*(v1) and Ts*PTA*(v1) genes were codon-optimized to *S. cerevisiae* using the GeneArt Gene Synthesis service (ThermoFisher Scientific). Ts*PTA*(v2) and Ca*XPK*(v1) were codon-optimized to *Y. lipolytica* using GeneArt Gene Synthesis service and the open source web application ATGme [59], respectively. Ca*XPK*(v2) was codon-optimized using the ATGme web application by manual replacement of all possible codons in the gene present at a frequency ≤ 2% with their higher frequency counterparts. All the gene sequences used in the strain engineering are listed in Additional file 1: Table S4.

### Glucose batch fermentation in 1-L bioreactors

Frozen working-stocks of strains were patched onto a YPD plate and grown overnight at 30 °C. A 10-µL loopful of cells was removed from each plate and used to inoculate separate 250-mL baffled Erlenmeyer flasks with 50 mL of lipid production media. Inoculum flasks were cultured overnight at 30 °C with constant agitation of 200 rpm in a New Brunswick I26 incubator shaker, whereupon the OD_600_ was measured. A volume of each flask culture required to initiate its corresponding 1-L bioreactors at a T_0_ cell density of 0.4 OD_600_ was transferred to separate sterile conical tubes. Each conical tube was then brought to 50 mL with sterile diH_2_O and centrifuged at 4000 rpm for 3 min in an Eppendorf 5810 R centrifuge. The supernatant was decanted and the cells were then resuspended in 50 mL sterile diH_2_O. 25 mL of this washed inoculum was used to inoculate each of two 1-L working volume bioreactors (Dasgip, 1.2-L vessels) with medium consisting of: glucose (150 g/L), (NH_4_)_2_SO_4_ (0.5 g/L), KH_2_PO_4_ (4 g/L), yeast extract (3 g/L), Amberferm 4500 (50 mg/L), MgSO_4_·7H_2_O (2 g/L), D-biotin (1 mg/L), thiamine hydrochloride (12 mg/L), ZnSO_4_·7H_2_O (20 mg/L), MnSO_4_·H_2_O (180 mg/L), CoCl_2_·6H_2_O (0.03 mg/L), CuSO_4_·5H_2_O (0.2 mg/L), Na_2_MoO_4_·2H_2_O (160 mg/L), CaCl_2_·6H_2_O (800 mg/L), FeCl_3_·6H_2_O (75 mg/L), H_3_BO_3_ (40 mg/L). Process parameters included a pH control at 3.5 automatically adjusted with 10 N sodium hydroxide, a temperature of 30 °C, aeration at 0.3 vvm air, and agitation controlled at 1000 rpm. A sample of 10 mL was taken from each culture once per day. The samples were stored at 4 °C after each harvest until analyzed. For all time-points, broth analysis was conducted via HPLC. Total dry cell weight (DCW) and total lipid content were measured gravimetrically by a two-phase solvent extraction. Cell-specific lipid productivities were calculated once the strains reached lipogenesis and their growth had slowed (day 2–day 5).

### Gravimetric measurement of dry cell weight (DCW) and total lipid content using a two-phase solvent extraction

Broth volume from each harvested culture sample was added to a separate pre-weighed 2-mL screw-cap microfuge tube (USA Scientific, 1420–8799) to achieve a dried cell mass between 15 and 20 mg. Samples were washed twice with deionized H_2_O and centrifuged at 21,130 × *g* for 2 min. Pelleted cells were then resuspended in 200 µL of deionized H_2_O, frozen at − 80 °C for 30 min, and freeze-dried overnight. Each tube was weighed to obtain the DCW. To each freeze-dried sample and three blank microfuge tubes, 400 mg of glass beads (Sigma, G8772) and 400 µL of a 1.5:1 CPME:MeOH (cyclopentyl methyl ether:methanol) solution was added. Under maximum agitation, samples were then bead-beaten (BioSpec Mini-BeadBeater 8) for 2 min and allowed to cool to room temperature. After having cooled, 640 µL of CPME followed by 640 µL of 10% (w/v) CaCl_2_·6H_2_O were added to each sample and vortexed. Samples were then centrifuged for 2 min at 21,130 × *g*, creating two distinct layers. 660 µL (75% of calculated volume) of the top layer, containing CPME and lipid, was transferred to a pre-weighed glass vial. Dispensed samples were evaporated under compressed air until no visual solvent remained and then lyophilized overnight for total solvent removal. The remaining lipid was weighed and corrected by subtracting the average residual mass measured in the blank samples.

### HPLC analysis

The extracellular concentrations of glucose, citrate and polyols (erythritol, arabitol and mannitol) were determined by high-performance liquid chromatography analysis. To that end, a 1-mL broth sample was filtered through a 0.2-mm syringe filter and analyzed using an Aminex HPX-87H column (300 mm × 7.8 mm) (Bio-Rad) on an Agilent 1260 Infinity II HPLC equipped with a refraction index detector (Agilent Technologies). The column was eluted with 5 mM H_2_SO_4_ at a flow rate of 0.6 mL min^−1^ at 45 °C for 25 min. The eluents were determined by comparing peak retention times to those of known standard substances, and the amounts were quantified by comparing the peak area of the analyte to the peak area of the standard substance at known concentrations.

## Supplementary Information

**Additional file 1.** Additional figures and tables.

## Data Availability

All data generated during this study are included in this published article and its Additional file. The publicly available *Y. lipolytica* transcriptomics data analyzed in this article were accessed from the NCBI GEO database (Series Accession Numbers GSE35445, GSE35446 and GSE35447).
