# Supplementary material for: Increasing lipid yield in Yarrowia lipolytica through phosphoketolase and phosphotransacetylase expression in a phosphofructokinase deletion strain
Source: Biotechnol Biofuels. 2021 May 4;14:113. doi: 10.1186/s13068-021-01962-6 (PMC8094482; doi:10.1186/s13068-021-01962-6)
Supplement: Supplementary file 1 — Additional file 1. Additional figures and tables. [file 13068_2021_1962_MOESM1_ESM.docx]

Supplementary material

**Supplementary Fig. S1A. Pathway depicting triolein synthesis from acetyl-CoA in *Y. lipolytica*.** A total of 27 Acetyl-CoA, 48 NADPH and 1 Glycerol-3-P are used to make 1 Triolein.

*
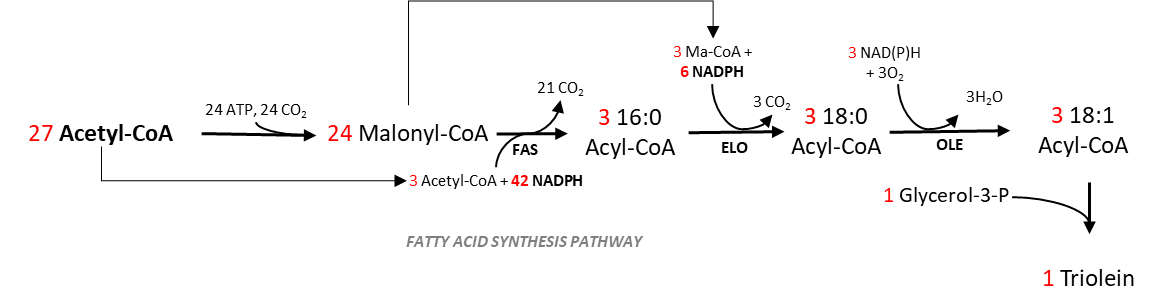
*

*
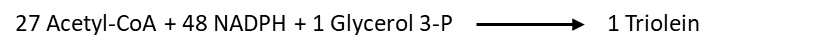
*

**Supplementary Fig. S1B.** **Acetyl-CoA production in *Y. lipolytica* using the native pathway.** Glycolysis, partial TCA cycle reactions and ATP:Citrate Lyase (ACL) are involved in the production of acetyl-CoA. NADPH requirement is fulfilled by the pentose phosphate pathway.

*
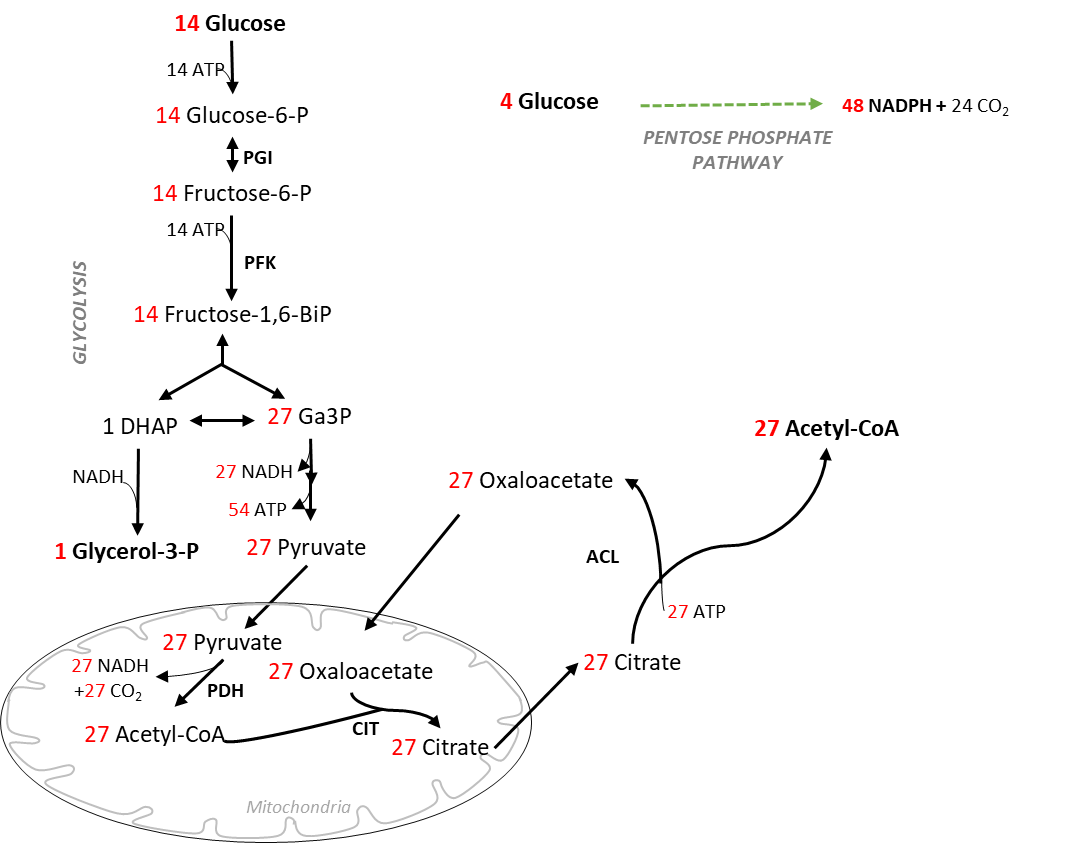
*

18 Glucose

1 Glycerol 3-P + 27 Acetyl-CoA + 48 NADPH

**Supplementary Fig. S1C. Acetyl-CoA production in *Y. lipolytica* using the Xpk/Pta/*Δpfk1* pathway.** Xpk/Pta pathway ties acetyl-CoA production to NADPH production, reducing the dependency on pentose phosphate pathway for NADPH production. *PFK1* deletion partially disables glycolysis forcing carbon flux through the heterologous pathway. Glyceraldehyde 3-phosphate (Ga3P) made by the Xpk/Pta pathway can still be metabolized via glycolysis to produce acetyl-CoA and glycerol 3-phosphate. This route utilizes 15.667 molecules of glucose to make 1 molecule of triolein resulting in a lipid yield of 0.31 g/g. Ga3P can also be recycled through the Xpk/Pta pathway via the fructose 1,6-bisphosphatase step but a complete recycle of all Ga3P would result in a redox imbalance (not shown). The Ga3P flux could potentially be split between the two routes (recycle through Xpk/Pta and glycolysis). An appropriate flux distribution could have a balanced redox and a positive impact on lipid yield.

*
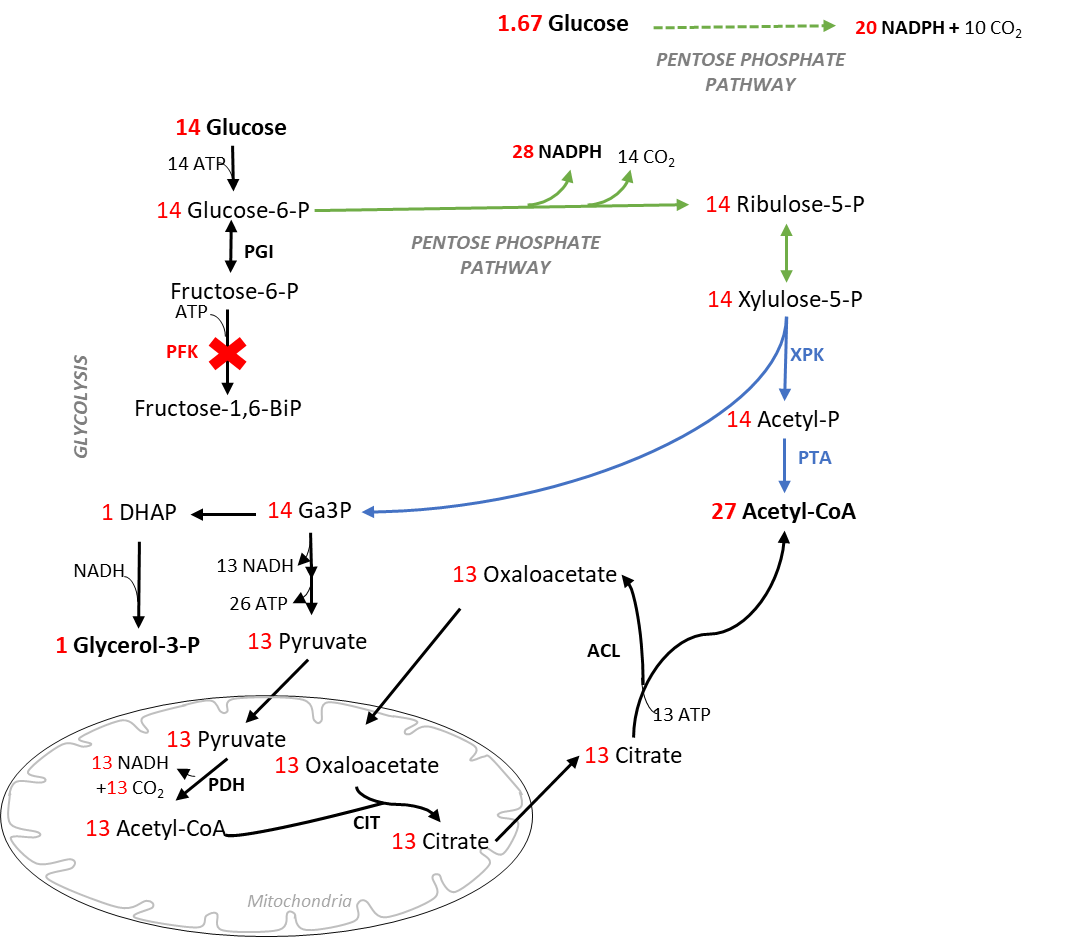
*

15.667 Glucose

1 Glycerol 3-P + 27 Acetyl-CoA + 48 NADPH

**Table S1: List of *PTA* and *XPK* genes tested in *Y. lipolytica***

**Table S1.1: List of *PTA* genes tested in *Y. lipolytica.* CO – codon optimized.**

| **Donor Organism** | **Accession #** | **Variants tested** |
| --- | --- | --- |
| *Methanosarcina thermophila* | P38503 | - Native |
| *Methanosarcina barkeri* | Q46BI0 | - Native |
| *Methanosarcina acetivorans* | Q8TK18 | - Native |
| *Bacillus subtilis* | P39646 | - Native - CO to *S. cerevisiae* - CO to *Y. lipolytica* |
| *Clostridium acetobutylicum* | P71103 | - Native |
| *Thermoanaerobacterium saccharolyticum* | I3VW55 | - Native - CO to *S. cerevisiae* - CO to *Y. lipolytica* |
| *Aphanomyces astaci* | W4HBJ2 | - CO to *S. cerevisiae* |
| *Aphanomyces invadans* | A0A024UQB7 | - CO to *S. cerevisiae* |
| *Auxenochlorella protothecoides* | A0A087SL07 | - CO to *S. cerevisiae* |
| *Beauveria bassiana* | A0A0A2W136 | - CO to *S. cerevisiae* |
| *Chlamydomonas reinhardtii* | A8IQQ1 | - CO to *S. cerevisiae* |
| *Guillardia theta* | L1JP48 | - CO to *S. cerevisiae* |
| *Helicosporidium sp.* | A0A059LKK9 | - CO to *S. cerevisiae* |
| *Perkinsus marinus* | C5LE15 | - CO to *S. cerevisiae* |
| *Phytophthora parasitica* | W3A4X6 | - CO to *S. cerevisiae* |
| *Phytophthora ramorum* | H3GP49 | - CO to *S. cerevisiae* |
| *Phytophthora sojae* | G4ZCU5 | - CO to *S. cerevisiae* |
| *Pythium ultimum* | K3X6T0 | - CO to *S. cerevisiae* |
| *Saprolegnia diclina* | T0PRY0 | - CO to *S. cerevisiae* |
| *Selaginella moellendorffii* | D8STZ3 | - CO to *S. cerevisiae* |
| *Volvox carteri* | D8TQ82 | - CO to *S. cerevisiae* |

**Table S1.2: List of *XPK* genes tested in *Y. lipolytica.* CO – codon optimized.**

| **Donor Organism** | **Accession** | **Variants tested** |
| --- | --- | --- |
| *Rhodosporidium toruloides* | M7WGA7 | - Native - CO to *S. cerevisiae* |
| *Aspergillus niger* | A2QDB0 | - Native - CO to *S. cerevisiae* |
| *Penicillium chrysogenum* | B6HBY3 | - Native |
| *Trichoderma reesei* | A0A024S8D5 | - Native - CO to *S. cerevisiae* |
| *Lactobacillus curvatus* | EHE86779 | - Native - CO to *S. cerevisiae* - CO to *Y. lipolytica* |
| *Aspergillus nidulans* | C8V9F5 | - Native - CO to *S. cerevisiae* |
| *Bifidobacterium adolescentis* | Q6R2R0 | - CO to *S. cerevisiae* |
| *Leuconostoc mesenteroides* | A0A5M8XD05 | - CO to *S. cerevisiae* |
| *Lactococcus lactis* | Q9CFH4 | - CO to *S. cerevisiae* |
| *Lactobacillus graminis* | A0A5J6Z6A6 | - CO to *S. cerevisiae* |
| *Lactobacillus fuchuensis* | A0A0R1RRQ4 | - CO to *S. cerevisiae* |
| *Lactobacillus concavus* | A0A0R1VX71 | - CO to *S. cerevisiae* |
| *Lactobacillus dextrinicus* | A0A0R2BTA3 | - CO to *S. cerevisiae* - CO to *Y. lipolytica* |
| *Lactobacillus ceti* | A0A0R2KG92 | - CO to *S. cerevisiae* - CO to *Y. lipolytica* |
| *Bifidobacterium breve* | D4BNU9 | - CO to *S. cerevisiae* |
| *Leuconostoc lactis* | KQB80457 | - CO to *S. cerevisiae* |
| *Oenococcus oeni* | A0NKP8 | - CO to *S. cerevisiae* |
| *Clostridium acetobutylicum* | Q97JE3 | - CO to *S. cerevisiae* - CO to *Y. lipolytica* |
| *Clostridium carboxidivorans* | C6PNC7 | - CO to *S. cerevisiae* |
| *Streptococcus pantholopis* | A0A172Q961 | - CO to *S. cerevisiae* |
| *Streptococcus thermophilus* | WP_049554686 | - CO to *S. cerevisiae* - CO to *Y. lipolytica* |

**Table S2.1: Steps involved in the construction of strain NS1475**

| **Strains** | **Genotype** | **# of isolates screened** | **Screening assay** |
| --- | --- | --- | --- |
| NS1047 | *Δpfk1* | 7 | Colony PCR followed by growth on YNB plates |
| NS1281 | *Δpfk1,* Ca*XPK*(v1) | 10 | Ferric hydroxamate assay |
| NS1292 | *Δpfk1,* 2xCa*XPK*(v1) | 10 | Ferric hydroxamate assay |
| NS1322 | *Δpfk1,* 3xCa*XPK*(v1) | 10 | Ferric hydroxamate assay |
| NS1341 | *Δpfk1,* 3xCa*XPK*(v1), Bs*PTA*(v1) | 10 | DTNB assay |
| NS1352 | *Δpfk1,* 3xCa*XPK*(v1), 2xBs*PTA*(v1) | 8 | DTNB assay |
| NS1420 | *Δpfk1,* 3xCa*XPK*(v1), 2xBs*PTA*(v1), Ts*PTA*(v2) | 10 | DTNB assay |
| NS1457 | *Δpfk1,* 3xCa*XPK*(v1), 2xBs*PTA*(v1), Ts*PTA*(v2), Ca*XPK*(v2) | 46 | Growth and lipid assay on lipid production media, microscopy |
| NS1475 | *Δpfk1,* 3xCa*XPK*(v1), 2xBs*PTA*(v1), Ts*PTA*(v2), 2xCa*XPK*(v2) | 90 | Growth and lipid assay on lipid production media, microscopy |

**Table S2.2: Steps involved in the construction of strains 1656 and 1657**

| Strains | Genotype | # of isolates screened | Screening assay |
| --- | --- | --- | --- |
| NS1600 | *Δpfk1*, Ts*PTA*(v2) | 10 | DTNB assay |
| NS1618 | *Δpfk1*, Ts*PTA*(v2), Ca*XPK*(v2) | 12 | Ferric hydroxamate assay |
| NS1636 | *Δpfk1*, Ts*PTA*(v2), 2xCa*XPK*(v2) | 76 | Growth and lipid assay on lipid production media |
| NS1656-57 | *Δpfk1*, Ts*PTA*(v2), 3xCa*XPK*(v2) | 52 | Growth and lipid assay on lipid production media |

**Fig. S2.A: Distribution plot of rare codons (≤1%) among two groups of genes, the highest expressors and moderate-low expressors****.** To understand codon usage among the highest expressed genes in *Y. lipolytica* we analyzed the published dataset generated by Ochoa-Estopier *et al.* 2014. In that experiment, *Y. lipolytica* W29 was cultivated under chemostat and decelerostat (Dstat) conditions. We used the sum of the transcript levels measured across all the timepoints during the experiment to rank ~6500 genes. We compared two groups of genes, 20 of the highest expressed genes (ranks 1-20) with 20 genes of moderate-low expression (ranked ~3000). We recorded the number of codons present at a frequency ≤ 1% (rare codons) for each of these genes using ATGme. The distribution plot of the rare codons across these two groups of genes is shown below. Since the trend suggested that rare codons may be detrimental for expression, we tested this theory by replacing all possible codons at frequency ≤1.5% and ≤2% with their higher frequency counterparts (Fig.S2 B).

**
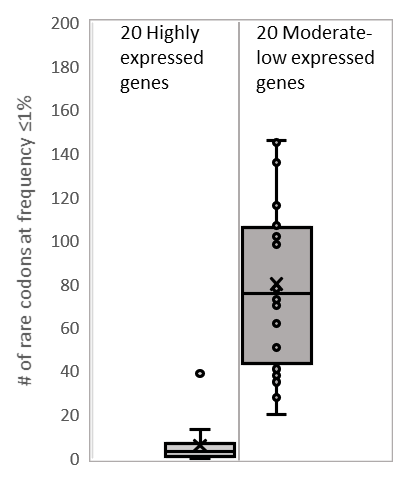
**

**Fig. S2.B: Testing differently codon optimized versions of Ca*XPK* in *Y. lipolytica*.** Genes were either integrated into the chromosome or expressed on a replicating plasmid.

**Table S3: *PFK* deletion cassette:**

| 1 | 47-bp sequence immediately upstream of *PFK* | CGCAAAACAGACGGACACTGAACCCCCCGCGCTTCAAAACACCGACA |
| --- | --- | --- |
| 2 | *Streptomyces noursei* *nat* gene, conferring nourseothricin resistance codon optimized for expression in yeast | ATGACCACTCTGGATGACACCGCTTACCGATACCGAACTTCCGTTCCTGGCGATGCCGAGGCTATTGAGGCTCTGGATGGATCTTTCACCACTGACACCGTTTTCCGAGTGACCGCTACTGGCGACGGCTTCACCCTGCGAGAGGTGCCTGTCGACCCTCCTCTCACCAAGGTTTTCCCTGACGATGAGTCGGACGATGAGTCTGACGCTGGAGAGGACGGCGACCCTGACTCTCGAACTTTCGTGGCTTACGGCGACGATGGAGACCTGGCCGGCTTTGTGGTCGTTTCTTACTCCGGATGGAACCGACGACTGACCGTGGAGGACATCGAGGTCGCTCCTGAGCACCGAGGTCATGGTGTCGGACGAGCTCTGATGGGTCTCGCTACTGAGTTCGCTCGAGAGCGAGGTGCTGGCCACCTGTGGCTCGAGGTCACCAACGTTAACGCCCCTGCTATTCATGCCTACCGACGAATGGGTTTTACCCTGTGTGGCCTCGATACTGCCCTGTACGACGGAACCGCTTCCGATGGAGAGCAGGCCCTCTACATGTCGATGCCCTGCCCTTAA |
| 3 | *S. cerevisiae CYC1* terminator | ACAGGCCCCTTTTCCTTTGTCGATATCATGTAATTAGTTATGTCACGCTTACATTCACGCCCTCCTCCCACATCCGCTCTAACCGAAAAGGAAGGAGTTAGACAACCTGAAGTCTAGGTCCCTATTTATTTTTTTTAATAGTTATGTTAGTATTAAGAACGTTATTTATATTTCAAATTTTTCTTTTTTTTCTGTACAAACGCGTGTACGCATGTAACATTATACTGAAAACCTTGCTTGAGAAGGTTTTGGGACGCTCGAAGGCTTTAATTTGC |
| 4 | *Y. lipolytica TEF1* promoter | AGAGACCGGGTTGGCGGCGCATTTGTGTCCCAAAAAACAGCCCCAATTGCCCCAATTGACCCCAAATTGACCCAGTAGCGGGCCCAACCCCGGCGAGAGCCCCCTTCTCCCCACATATCAAACCTCCCCCGGTTCCCACACTTGCCGTTAAGGGCGTAGGGTACTGCAGTCTGGAATCTACGCTTGTTCAGACTTTGTACTAGTTTCTTTGTCTGGCCATCCGGGTAACCCATGCCGGACGCAAAATAGACTACTGAAAATTTTTTTGCTTTGTGGTTGGGACTTTAGCCAAGGGTATAAAAGACCACCGTCCCCGAATTACCTTTCCTCTTCTTTTCTCTCTCTCCTTGTCAACTCACACCCGAAATCGTTAAGCATTTCCTTCTGAGTATAAGAATCATTCAAA |
| 5 | Herpes Simplex Virus thymidine kinase gene *hsvTDK* codon-optimized for expression in *Y. lipolytica* | ATGGCTTCTTACCCTTGCCACCAGCACGCTTCCGCTTTTGACCAGGCCGCCCGATCCCGAGGACACTCCAACCGACGAACCGCTCTGCGACCCCGACGACAGCAGGAGGCTACCGAGGTTCGACTGGAGCAGAAGATGCCTACTCTGCTCCGAGTGTACATCGACGGACCCCACGGTATGGGCAAGACCACTACCACTCAGCTGCTCGTCGCCCTGGGTTCGCGAGATGACATTGTTTACGTGCCTGAGCCCATGACCTACTGGCAGGTTCTCGGAGCTTCTGAGACTATCGCCAACATCTACACCACTCAGCATCGACTGGACCAGGGAGAGATCTCCGCTGGAGATGCCGCTGTGGTCATGACCTCGGCCCAGATTACTATGGGCATGCCTTACGCTGTCACCGACGCTGTTCTGGCTCCTCACATCGGTGGAGAGGCTGGATCTTCCCATGCTCCTCCTCCTGCTCTGACCCTCATCTTCGATCGACACCCTATTGCCGCTCTGCTCTGTTACCCCGCCGCTCGATACCTGATGGGCTCTATGACCCCTCAGGCCGTGCTGGCTTTTGTCGCCCTCATCCCTCCCACCCTGCCTGGTACTAACATTGTGCTGGGTGCTCTCCCTGAGGACCGACATATCGATCGACTCGCTAAGCGACAGCGACCTGGAGAGCGACTGGACCTCGCTATGCTGGCCGCTATTCGACGAGTGTACGGCCTGCTCGCTAACACCGTCCGATACCTCCAGGGTGGTGGATCGTGGCGAGAGGACTGGGGACAGCTGTCTGGTACCGCTGTGCCTCCTCAGGGTGCTGAGCCTCAGTCCAACGCTGGACCTCGACCCCACATCGGTGACACCCTGTTCACTCTCTTTCGAGCTCCTGAGCTGCTCGCTCCTAACGGCGACCTGTACAACGTCTTCGCCTGGGCTCTGGATGTTCTCGCCAAGCGACTCCGACCTATGCACGTCTTTATTCTGGACTACGATCAGTCGCCCGCTGGATGTCGAGATGCCCTGCTCCAGCTCACCTCTGGCATGGTTCAGACTCATGTGACCACTCCTGGATCCATCCCCACCATTTGCGATCTGGCTCGAACTTTCGCCCGAGAGATGGGAGAGGCCAACTAA |
| 6 | *Y. lipolytica TEF1* terminator | GCTGCTTGTACCTAGTGCAACCCCAGTTTGTTAAAAATTAGTAGTCAAAAACTTCTGAGTTAGAAATTTGTGAGTGTAGTGAGATTGTAGAGTATCATGTGTGTCCGTAAGTGAAGTGTTATTGACTCTTAGTTAGTTTATCTAGTACTCGTTTAGTTGACACTGATCTAGTATTTTACGAGGCGTATGACTTTAGCCAAGTGTTGTACTTAGTCTTCTCTCCAAACATGAGAGGGCTCTGTCACTCAGTCGGCCTATGGGTGAGATGGCTTGGTGAGATCTTTCGATAGTCTCGTCAAGATGGTAGGATGATGGGGGAATACATTACTGCTCTCGTCAAGGAAACCACAATCAGATCACACCATCCTCCATGGTATCCGATGACTCTCTTCTCCACAGT |
| 7 | 450-bp sequence corresponding to nucleotides -636 to -187 upstream of *PFK* | CTTTTGATCCGATGGTTACTTTTTATGTTCTATTTTACATTAGCGTGGAAATAGACCATGCCATCTTTGGCACCCCGGAAAAACTTGATCCAATAGAGTTGTTGGGTGGAGCTAGTGACTGGCGGCAATTGGAGAGCTTCTAGAAGACGAACCAGGAGCCCGATAGGAACTCCGTTGGCGTGAGTCGGCCCCCCAGTAGCAATTCGAATCACGTGACGTGGAGTTTTCCCTCGCCCGCGTTCCTGGATTGTCCCGGTGTGACGAGGCCGACTGGATTTGATCACCCAACCCCACACGACGCATAATGTAAATGTATCATCATACAGTACATGCCCGAGTCTAATGATTGGCTGGTTCACGGGGACCGGGAGCGTCCAGTGGGCCGTATGGCGGTGGTTCAAACACCTCAGATCAGCTCACTATCGGCTGAGACAATCCTTAACTGTTGGT |
| 8 | 621-bp sequence immediately downstream of *PFK* | GGGGTTTGGTGTTGGAAATTAGGATATCTATTTGATTAATGTAGCTTGGTTTTGGACAAGAATGCTGATTGATACATCCGGTATCACTTGTATACAACGTAGGGGGCGGTGTATGGGTGAAAATTTCGAAAGACTGAGAATCGAACTGGGAGGTTTTTAAACTCCTGAAACTGTAGTTGGTGAACGAGCCGAGGAGTATTTTAGACCCGAAACATTTGGTGCAACCAGATACGCCAGGTAAACAATCTACACTACACTACATGGGACGAGGGTTGAAAAGGTGGTGACAGGGATATTGCAGATGGGATTCTCGTGATGATGAGCCGTCAGCGTCTCGGCTTGTCTTTGTGTTCAACGGCTTGTCTACGTCTGCTCGTATCCACCTGTCATGTTGGTTGTTCCATGCTTATAGGCCTGTCTGTTGGGGGCAGTAAGGTTTTGAGAAGTGTGTAGTATGGGGATGTATTATCTTGGGGTTTTGGTTGAGATATTAGTAATCTGAGAGTTTGAGGGTATTTCTGGCGGTTTGTGTATATAAGTCCGGCTTTTTCCAAGTCGATATCTCCTTTTGTTGTTGTTTTGGGGCCGCTTTTATTTCTTCCAGGGCTGTAGGCGAAATTC |

**Table S3.1: Oligonucleotide primers used to amplify cassette in two overlapping fragments**:

| Primer | Description | Sequence |
| --- | --- | --- |
| NP2782 | 5’ fragment forward primer | CGCAAAACAGACGGACACTGAACCCCCCGCGCTTCAAAACACCGACAATGACCACTCTGGATGACACC |
| NP356 | 5’ fragment reverse primer | TCTCCATCGGAAGCGGTTCCGTC |
| NP355 | 3’ fragment forward primer | AACTTCCGTTCCTGGCGATGCCG |
| NP3531 | 3’ fragment reverse primer | GAATTTCGCCTACAGCCCTGG |

**Table S4: *pta* or *xpk* expression cassette:**

**Table S4.1: *pta* expression cassette used in Section 3.1 for finding functional *pta* in *Y. lipolytica***

| 1 | *S. cerevisiae FBA1* promoter | GATCCAACTGGCACCGCTGGCTTGAACAACAATACCAGCCTTCCAACTTCTGTAAATAACGGCGGTACGCCAGTGCCACCAGTACCGTTACCTTTCGGTATACCTCCTTTCCCCATGTTTCCAATGCCCTTCATGCCTCCAACGGCTACTATCACAAATCCTCATCAAGCTGACGCAAGCCCTAAGAAATGAATAACAATACTGACAGTACTAAATAATTGCCTACTTGGCTTCACATACGTTGCATACGTCGATATAGATAATAATGATAATGACAGCAGGATTATCGTAATACGTAATAGTTGAAAATCTCAAAAATGTGTGGGTCATTACGTAAATAATGATAGGAATGGGATTCTTCTATTTTTCCTTTTTCCATTCTAGCAGCCGTCGGGAAAACGTGGCATCCTCTCTTTCGGGCTCAATTGGAGTCACGCTGCCGTGAGCATCCTCTCTTTCCATATCTAACAACTGAGCACGTAACCAATGGAAAAGCATGAGCTTAGCGTTGCTCCAAAAAAGTATTGGATGGTTAATACCATTTGTCTGTTCTCTTCTGACTTTGACTCCTCAAAAAAAAAAAATCTACAATCAACAGATCGCTTCAATTACGCCCTCACAAAAACTTTTTTCCTTCTTCTTCGCCCACGTTAAATTTTATCCCTCATGTTGTCTAACGGATTTCTGCACTTGATTTATTATAAAAAGACAAAGACATAATACTTCTCTATCAATTTCAGTTATTGTTCTTCCTTGCGTTATTCTTCTGTTCTTCTTTTTCTTTTGTcatatataaccataaccaagtaatacatattcaaa |
| --- | --- | --- |
| 2 | *E. coli hph* gene, conferring hygromycin B resistance (GenBank: AEJ60084.1) codon-optimized for expression in *Y. lipolytica* (Genscript) | ATGAAGAAGCCCGAGCTGACCGCTACCTCTGTTGAGAAGTTCCTGATTGAGAAGTTTGATTCCGTTTCCGACCTGATGCAGCTGTCCGAGGGCGAGGAGTCTCGAGCCTTCTCCTTTGACGTGGGCGGACGAGGTTACGTTCTGCGAGTGAACTCGTGTGCCGACGGCTTCTACAAGGATCGATACGTCTACCGACACTTTGCTTCTGCCGCTCTGCCCATCCCTGAGGTTCTCGACATTGGCGAGTTCTCTGAGTCCCTCACCTACTGCATCTCTCGACGAGCTCAGGGAGTCACCCTGCAGGACCTCCCTGAGACTGAGCTGCCTGCTGTCCTCCAGCCTGTTGCTGAGGCCATGGACGCTATCGCTGCTGCTGATCTGTCCCAGACCTCGGGTTTCGGCCCCTTTGGACCTCAGGGAATTGGACAGTACACCACTTGGCGAGACTTCATCTGTGCTATTGCCGATCCTCACGTCTACCATTGGCAGACCGTTATGGACGATACTGTGTCGGCTTCTGTCGCTCAGGCTCTGGACGAGCTGATGCTCTGGGCCGAGGATTGCCCCGAGGTTCGACACCTGGTGCATGCTGACTTCGGTTCCAACAACGTTCTCACCGACAACGGCCGAATCACTGCCGTGATTGACTGGTCCGAGGCTATGTTTGGCGACTCGCAGTACGAGGTGGCCAACATCTTCTTTTGGCGACCCTGGCTGGCTTGTATGGAGCAGCAGACCCGATACTTCGAGCGACGACATCCTGAGCTCGCTGGATCCCCTCGACTGCGAGCTTACATGCTCCGAATTGGTCTGGACCAGCTCTACCAGTCGCTGGTGGATGGCAACTTTGACGATGCTGCCTGGGCTCAGGGACGATGTGACGCCATCGTGCGATCTGGCGCTGGAACCGTCGGACGAACTCAGATTGCCCGACGATCCGCTGCTGTCTGGACCGACGGATGCGTGGAGGTCCTGGCTGATTCGGGTAACCGACGACCCTCTACTCGACCTCGAGCTAAGGAGTAA |
| 3 | *S. cerevisiae FBA1* terminator | gttaattcaaattaattgatatAGTTTTTTAATGAGTATTGAATCTGTTTAGAAATAATGGAATATTATTTTTATTTATTTATTTATATTATTGGTCGGCTCTTTTCTTCTGAAGGTCAATGACAAAATGATATGAAGGAAATAATGATTTCTAAAATTTTACAACGTAAGATATTTTTACAAAAGCCTAGCTCATCTTTTGTCA |
| 4 | *Y. lipolytica EXP1* promoter | GAGTTTGGCGCCCGTTTTTTCGAGCCCCACACGTTTCGGTGAGTATGAGCGGCGGCAGATTCGAGCGTTTCCGGTTTCCGCGGCTGGACGAGAGCCCATGATGGGGGCTCCCACCACCAGCAATCAGGGCCCTGATTACACACCCACCTGTAATGTCATGCTGTTCATCGTGGTTAATGCTGCTGTGTGCTGTGTGTGTGTGTTGTTTGGCGCTCATTGTTGCGTTATGCAGCGTACACCACAATATTGGAAGCTTATTAGCCTTTCTATTTTTTCGTTTGCAAGGCTTAACAACATTGCTGTGGAGAGGGATGGGGATATGGAGGCCGCTGGAGGGAGTCGGAGAGGCGTTTTGGAGCGGCTTGGCCTGGCGCCCAGCTCGCGAAACGCACCTAGGACCCTTTGGCACGCCGAAATGTGCCACTTTTCAGTCTAGTAACGCCTTACCTACGTCATTCCATGCATGCATGTTTGCGCCTTTTTTCCCTTGCCCTTGATCGCCACACAGTACAGTGCACTGTACAGTGGAGGTTTTGGGGGGGTCTTAGATGGGAGCTAAAAGCGGCCTAGCGGTACACTAGTGGGATTGTATGGAGTGGCATGGAGCCTAGGTGGAGCCTGACAGGACGCACGACCGGCTAGCCCGTGACAGACGATGGGTGGCTCCTGTTGTCCACCGCGTACAAATGTTTGGGCCAAAGTCTTGTCAGCCTTGCTTGCGAACCTAATTCCCAATTTTGTCACTTCGCACCCCCATTGATCGAGCCCTAACCCCTGCCCATCAGGCAATCCAATTAAGCTCGCATTGTCTGCCTTGTTTAGTTTGGCTCCTGCCCGTTTCGGCGTCCACTTGCACAAACACAAACAAGCATTATATATAAGGCTCGTCTCTCCCTCCCAACCACACTCACTTTTTTGCCCGTCTTCCCTTGCTAACACAAAAGTCAAGAACACAAACAACCACCCCAACCCCCTTACACACAAGACATATCTACAGCA |
| 5 | *pta genes* | [gene_of_interest] |
| 6 | *Y. lipolytica CYC1* terminator | GCGTCTACAACTGGACCCTTAGCCTGTATATATCAATTGATTATTTAAAGATTTGGTCGGTAGGCGGTTCGTATTGTACAATGGGATCTGTTACTGAGGTGGATCTACCCAACTTGCGAGATTCAATTGCGAGATTCAATCGCGAGATTCAATTGCGAGAATCAGTTGCGAGTTGTTCTAACACTCAGCTTCTACGAGCGCTTGTATTAGGACGAGTGATACTCCGTGGGGCGACGGCTTCTCTTGCGTCTTCTGTTGTATTCTTTCTTACACTATCGTCCATCTCCAACCACCTCGTAC |

**Table S4.2: Replicating plasmid containing the *xpk* expression cassette used in Section 3.1 for finding functional *xpk* in *Y. lipolytica***

| 1 | Autonomous replicating sequence ARS68 | AGCTAGCTCGTCGTGTTCAGGAACTGTTCGATGGTTCGGAGAGAGTCGCCGCCCAGAACATACGCGCACCGATGTCAGCAGACAGCCTTATTACAAGTACAGTATGTACATACTACTGTATATTCAAGCAAGTATATCCGTAGGGTGCGGGTGATTTGGATCTAAGGTTCGTACTCAACACTCACGAGCAGCTTGCCTATGTTACATCCTTTTATCAGACATAACATAATTGGAGTTTACTTACACACGGGGTGTACCTGTATGAGCACCACCTACAATTGTAGCACTGGTACTTGTACAAAGAATTTATTCGTACGAATCACAGGAACGGCCGCCCTCACCGAACCAGCGAATACCTCAGCGGTCCCCTGCAGTGACTCAACAAAGCGATATGAACATCTTGCGATGGTATCCTGCTGATAGTTTTTACTGTACAAACACCTGTGTAGCTCCTTCTAGCATTTTTAAGTTATTCACACCTCAAGGGGAGGGATAAATTAAATAAATTCCAAAAGCGAAGATCGAGAAACTAAATTAAAATTCCAAAAACGAAGTTGGAACACAACCCCCCGAAAAAAAACAACAAGCAAAAAACCCAACAAAATAAACAAAAACAAAATAAATATATAACAAAATAAATATATAACTACCAGTATCTGACTAAAAGTTCAAATACTCGTACTTACAACAAATAGAAATGAGCCGGCCAAAATTCTGCAGAAAAAATTTTCAAACAAGTACTTTCAAACAAGTACTGGTATAATTAAATTAAAAAACACATCAAAGTATCATAACGTTAGTTATTTTATTTTATTTAATAAAAGAAAACAACAAAATGGGCTCAAAACTTTCAACTTATACG |
| --- | --- | --- |
| 2 | Centromere CEN1-1 | ATACATACCAAATAACAATTTAGTATTTATCTAAGTGCTTTTCGTAGATAATGGAATACAAATGGATATCCAGAGTATACACATGGATAGTATACACTGACACGACAATTCTGTATCTCTTTATGTTAACTACTGTGAGGCGTTAAATAGAGCTTGATATATAAAATGTTACATTTCACAGTCTGAACTTTTGCAGATTACCTAATTTGGTAAGATATTAATTATGAACTGAAAGTTGATG |
| 3 | *Arxula adeninivorans ADH1* promoter | tgcgtcggaacgggatatgcatTCCCCTAGTTTCGCCGCAGTGCAGAATCAGGCGGTTTCTTTGCACCACACCACATACGGAGGATGACGGGCATTATTGATGTTGAATAGTAACCTGATCGTGACTAGTATGACGGAACCCAACAGCAACAGCCGACCGTTTGTGAGCGTTTTTGCGGCCGGTCAGGCGAGTTTTTCCGGCCTGCCAATGGTCCTTCCGTACCCTTTACCCTGTACGCTGTACCTGCCACGGATAGGCCGTGCTCCACCTGCTCACTATGGTGGGTGCGGGGAAAACAACAGGCAGGCTCAATTGCTCTGCAAATGGGTTGAGGGGGTGATTGATGTCACTGGTACACCAACAGGGGAATGCTCGGCGTTGATTTTGGGCCACCTCTTTTGTTTGCCAGAGCTTGTCTCTATTGTCAAATTTAACGGTCTGCAACTGTTGCCCAAAATGGGACAATGATCCGATGCCTGCATAGACACCCTGCTTGAGGGTGCGATCGCCCTAATACGAGGCAAACCAAGTTTTCCAATTGACCTTCAATTGACGAGCGGTTGTTGCGACAGGGGACTGGAGTGCTACCTGTTTAGAGTTCAAATCCGTCACCCAGCATTGAAAGTTTTTCCCCGCATTGGATGATTGCAATGCCGCTAACCCGCTCATCCGCCAAAGTTCATAGTCCCACCCTGCCTCGACTTATCGGACCACATGGGGCTCCCTTATGCGCGCGCATATGGCGCTTGATTGCTTTTTGGTCAACGTTTGGGACAAATTTCCTTTGTTAAGGCGGACCCGCCAGCAGATACGAAGGTATAAATAGGGCTCACTTTCACCATCTTgtccattcaattgcaagactcaaaagtaata |
| 4 | *Streptomyces noursei* *Nat1* gene, conferring Nourseothricin resistance (GenBank: CAA51674.1) codon-optimized for expression in *Y. lipolytica* (Genscript) | ATGACCACTCTGGATGACACCGCTTACCGATACCGAACTTCCGTTCCTGGCGATGCCGAGGCTATTGAGGCTCTGGATGGATCTTTCACCACTGACACCGTTTTCCGAGTGACCGCTACTGGCGACGGCTTCACCCTGCGAGAGGTGCCTGTCGACCCTCCTCTCACCAAGGTTTTCCCTGACGATGAGTCGGACGATGAGTCTGACGCTGGAGAGGACGGCGACCCTGACTCTCGAACTTTCGTGGCTTACGGCGACGATGGAGACCTGGCCGGCTTTGTGGTCGTTTCTTACTCCGGATGGAACCGACGACTGACCGTGGAGGACATCGAGGTCGCTCCTGAGCACCGAGGTCATGGTGTCGGACGAGCTCTGATGGGTCTCGCTACTGAGTTCGCTCGAGAGCGAGGTGCTGGCCACCTGTGGCTCGAGGTCACCAACGTTAACGCCCCTGCTATTCATGCCTACCGACGAATGGGTTTTACCCTGTGTGGCCTCGATACTGCCCTGTACGACGGAACCGCTTCCGATGGAGAGCAGGCCCTCTACATGTCGATGCCCTGCCCTTAA |
| 5 | *S. cerevisiae CYC1* terminator | acaggccccttttcctttgtcgatatcatgtaattagttatgtcacgcttacattcacgccctcctcccacatccgctctaaccgaaaaggaaggagttagacaacctgaagtctaggtccctatttattttttttaatagttatgttagtattaagaacgttatttatatttcaaatttttcttttttttctgtacaaacgcgtgtacgcatgtaacattatactgaaaaccttgcttgagaaggttttgGGACGCTCGAAGGCTTTAATTTGC |
| 6 | *Y. lipolytica TEF1* promoter | agagaccgggttggcggcgcatttgtgtcccaaaaaacagccccaattgccccaattgaccccaaattgacccagtagcgggcccaaccccggcgagagcccccttctccccacatatcaaacctcccccggttcccacacttgccgttaagggcgtagggtactgcagtctggaatctacgcttgttcagactttgtactagtttctttgtctggccatccgggtaacccatgccggacgcaaaatagactactgaaaatttttttgctttgtggttgggactttagccaagggtataaaagaccaccgtccccgaattacctttcctcttcttttctctctctccttgtcaactcacacccgaaatcgtTAAGCATTTCCTTCTGAGTATAAGAATCATTCAAA |
| 7 | *xpk* gene of interest | [gene_of_interest] |
| 8 | *A. adeninivorans CYC1* terminator | GCGGTTTAGATTTTCCAATTGTAAATATATTACTGTACCATTCTGTACTAAATAACGTGTTTTTTATACTACTTCCTATCTATATTCTATATCGTTACTGGCATATATATATCGTTGCTGGAGGTCGAAGGTGAAATTTCACTTGCCTTTCTCTTCCCCGAGCCGCACGCCGCCATATCGTTATCTTGATTGAGCCCGACATCATGATCACTAACTGATTATGCTCCAGCAGGAGCAGGTTGTAGAGCTCCAAGATTTGCGAGGAGGTGGAGAGAATAGCACGGGACAGAGAGTACGATGG |

**Table S4.3: *pta* or *xpk* expression cassette used in the construction of strains NS1475 and NS1656-57**

| 1 | *Y. lipolytica TEF1* promoter | AGAGACCGGGTTGGCGGCGCATTTGTGTCCCAAAAAACAGCCCCAATTGCCCCAATTGACCCCAAATTGACCCAGTAGCGGGCCCAACCCCGGCGAGAGCCCCCTTCTCCCCACATATCAAACCTCCCCCGGTTCCCACACTTGCCGTTAAGGGCGTAGGGTACTGCAGTCTGGAATCTACGCTTGTTCAGACTTTGTACTAGTTTCTTTGTCTGGCCATCCGGGTAACCCATGCCGGACGCAAAATAGACTACTGAAAATTTTTTTGCTTTGTGGTTGGGACTTTAGCCAAGGGTATAAAAGACCACCGTCCCCGAATTACCTTTCCTCTTCTTTTCTCTCTCTCCTTGTCAACTCACACCCGAAATCGTTAAGCATTTCCTTCTGAGTATAAGAATCATTCAAA |
| --- | --- | --- |
| 2 | Herpes Simplex Virus thymidine kinase gene *hsvTDK* codon-optimized for expression in *Y. lipolytica* | ATGGCTTCTTACCCTTGCCACCAGCACGCTTCCGCTTTTGACCAGGCCGCCCGATCCCGAGGACACTCCAACCGACGAACCGCTCTGCGACCCCGACGACAGCAGGAGGCTACCGAGGTTCGACTGGAGCAGAAGATGCCTACTCTGCTCCGAGTGTACATCGACGGACCCCACGGTATGGGCAAGACCACTACCACTCAGCTGCTCGTCGCCCTGGGTTCGCGAGATGACATTGTTTACGTGCCTGAGCCCATGACCTACTGGCAGGTTCTCGGAGCTTCTGAGACTATCGCCAACATCTACACCACTCAGCATCGACTGGACCAGGGAGAGATCTCCGCTGGAGATGCCGCTGTGGTCATGACCTCGGCCCAGATTACTATGGGCATGCCTTACGCTGTCACCGACGCTGTTCTGGCTCCTCACATCGGTGGAGAGGCTGGATCTTCCCATGCTCCTCCTCCTGCTCTGACCCTCATCTTCGATCGACACCCTATTGCCGCTCTGCTCTGTTACCCCGCCGCTCGATACCTGATGGGCTCTATGACCCCTCAGGCCGTGCTGGCTTTTGTCGCCCTCATCCCTCCCACCCTGCCTGGTACTAACATTGTGCTGGGTGCTCTCCCTGAGGACCGACATATCGATCGACTCGCTAAGCGACAGCGACCTGGAGAGCGACTGGACCTCGCTATGCTGGCCGCTATTCGACGAGTGTACGGCCTGCTCGCTAACACCGTCCGATACCTCCAGGGTGGTGGATCGTGGCGAGAGGACTGGGGACAGCTGTCTGGTACCGCTGTGCCTCCTCAGGGTGCTGAGCCTCAGTCCAACGCTGGACCTCGACCCCACATCGGTGACACCCTGTTCACTCTCTTTCGAGCTCCTGAGCTGCTCGCTCCTAACGGCGACCTGTACAACGTCTTCGCCTGGGCTCTGGATGTTCTCGCCAAGCGACTCCGACCTATGCACGTCTTTATTCTGGACTACGATCAGTCGCCCGCTGGATGTCGAGATGCCCTGCTCCAGCTCACCTCTGGCATGGTTCAGACTCATGTGACCACTCCTGGATCCATCCCCACCATTTGCGATCTGGCTCGAACTTTCGCCCGAGAGATGGGAGAGGCCAACTAA |
| 3 | *S. cerevisiae FBA1* terminator | GTTAATTCAAATTAATTGATATAGTTTTTTAATGAGTATTGAATCTGTTTAGAAATAATGGAATATTATTTTTATTTATTTATTTATATTATTGGTCGGCTCTTTTCTTCTGAAGGTCAATGACAAAATGATATGAAGGAAATAATGATTTCTAAAATTTTACAACGTAAGATATTTTTACAAAAGCCTAGCTCATCTTTTGTCA |
| 4 | *Arxula adeninivorans ADH1* promoter | TGCGTCGGAACGGGATATGCATTCCCCTAGTTTCGCCGCAGTGCAGAATCAGGCGGTTTCTTTGCACCACACCACATACGGAGGATGACGGGCATTATTGATGTTGAATAGTAACCTGATCGTGACTAGTATGACGGAACCCAACAGCAACAGCCGACCGTTTGTGAGCGTTTTTGCGGCCGGTCAGGCGAGTTTTTCCGGCCTGCCAATGGTCCTTCCGTACCCTTTACCCTGTACGCTGTACCTGCCACGGATAGGCCGTGCTCCACCTGCTCACTATGGTGGGTGCGGGGAAAACAACAGGCAGGCTCAATTGCTCTGCAAATGGGTTGAGGGGGTGATTGATGTCACTGGTACACCAACAGGGGAATGCTCGGCGTTGATTTTGGGCCACCTCTTTTGTTTGCCAGAGCTTGTCTCTATTGTCAAATTTAACGGTCTGCAACTGTTGCCCAAAATGGGACAATGATCCGATGCCTGCATAGACACCCTGCTTGAGGGTGCGATCGCCCTAATACGAGGCAAACCAAGTTTTCCAATTGACCTTCAATTGACGAGCGGTTGTTGCGACAGGGGACTGGAGTGCTACCTGTTTAGAGTTCAAATCCGTCACCCAGCATTGAAAGTTTTTCCCCGCATTGGATGATTGCAATGCCGCTAACCCGCTCATCCGCCAAAGTTCATAGTCCCACCCTGCCTCGACTTATCGGACCACATGGGGCTCCCTTATGCGCGCGCATATGGCGCTTGATTGCTTTTTGGTCAACGTTTGGGACAAATTTCCTTTGTTAAGGCGGACCCGCCAGCAGATACGAAGGTATAAATAGGGCTCACTTTCACCATCTTGTCCATTCAATTGCAAGACTCAAAAGTAATA |
| 5 | *E. coli hph* gene, conferring hygromycin B resistance codon-optimized for expression in *Y. lipolytica* | ATGAAGAAGCCCGAGCTGACCGCTACCTCTGTTGAGAAGTTCCTGATTGAGAAGTTTGATTCCGTTTCCGACCTGATGCAGCTGTCCGAGGGCGAGGAGTCTCGAGCCTTCTCCTTTGACGTGGGCGGACGAGGTTACGTTCTGCGAGTGAACTCGTGTGCCGACGGCTTCTACAAGGATCGATACGTCTACCGACACTTTGCTTCTGCCGCTCTGCCCATCCCTGAGGTTCTCGACATTGGCGAGTTCTCTGAGTCCCTCACCTACTGCATCTCTCGACGAGCTCAGGGAGTCACCCTGCAGGACCTCCCTGAGACTGAGCTGCCTGCTGTCCTCCAGCCTGTTGCTGAGGCCATGGACGCTATCGCTGCTGCTGATCTGTCCCAGACCTCGGGTTTCGGCCCCTTTGGACCTCAGGGAATTGGACAGTACACCACTTGGCGAGACTTCATCTGTGCTATTGCCGATCCTCACGTCTACCATTGGCAGACCGTTATGGACGATACTGTGTCGGCTTCTGTCGCTCAGGCTCTGGACGAGCTGATGCTCTGGGCCGAGGATTGCCCCGAGGTTCGACACCTGGTGCATGCTGACTTCGGTTCCAACAACGTTCTCACCGACAACGGCCGAATCACTGCCGTGATTGACTGGTCCGAGGCTATGTTTGGCGACTCGCAGTACGAGGTGGCCAACATCTTCTTTTGGCGACCCTGGCTGGCTTGTATGGAGCAGCAGACCCGATACTTCGAGCGACGACATCCTGAGCTCGCTGGATCCCCTCGACTGCGAGCTTACATGCTCCGAATTGGTCTGGACCAGCTCTACCAGTCGCTGGTGGATGGCAACTTTGACGATGCTGCCTGGGCTCAGGGACGATGTGACGCCATCGTGCGATCTGGCGCTGGAACCGTCGGACGAACTCAGATTGCCCGACGATCCGCTGCTGTCTGGACCGACGGATGCGTGGAGGTCCTGGCTGATTCGGGTAACCGACGACCCTCTACTCGACCTCGAGCTAAGGAGTAA |
| 6 | *S. cerevisiae CYC1* terminator | ACAGGCCCCTTTTCCTTTGTCGATATCATGTAATTAGTTATGTCACGCTTACATTCACGCCCTCCTCCCACATCCGCTCTAACCGAAAAGGAAGGAGTTAGACAACCTGAAGTCTAGGTCCCTATTTATTTTTTTTAATAGTTATGTTAGTATTAAGAACGTTATTTATATTTCAAATTTTTCTTTTTTTTCTGTACAAACGCGTGTACGCATGTAACATTATACTGAAAACCTTGCTTGAGAAGGTTTTGGGACGCTCGAAGGCTTTAATTTGC |
| 7 | *Y. lipolytica TEF1* promoter | AGAGACCGGGTTGGCGGCGCATTTGTGTCCCAAAAAACAGCCCCAATTGCCCCAATTGACCCCAAATTGACCCAGTAGCGGGCCCAACCCCGGCGAGAGCCCCCTTCTCCCCACATATCAAACCTCCCCCGGTTCCCACACTTGCCGTTAAGGGCGTAGGGTACTGCAGTCTGGAATCTACGCTTGTTCAGACTTTGTACTAGTTTCTTTGTCTGGCCATCCGGGTAACCCATGCCGGACGCAAAATAGACTACTGAAAATTTTTTTGCTTTGTGGTTGGGACTTTAGCCAAGGGTATAAAAGACCACCGTCCCCGAATTACCTTTCCTCTTCTTTTCTCTCTCTCCTTGTCAACTCACACCCGAAATCGTTAAGCATTTCCTTCTGAGTATAAGAATCATTCAAA |
| 8 | *pta* or *xpk* gene of interest | [gene_of_interest] |
| 9 | *A. adeninivorans CYC1* terminator | GCGGTTTAGATTTTCCAATTGTAAATATATTACTGTACCATTCTGTACTAAATAACGTGTTTTTTATACTACTTCCTATCTATATTCTATATCGTTACTGGCATATATATATCGTTGCTGGAGGTCGAAGGTGAAATTTCACTTGCCTTTCTCTTCCCCGAGCCGCACGCCGCCATATCGTTATCTTGATTGAGCCCGACATCATGATCACTAACTGATTATGCTCCAGCAGGAGCAGGTTGTAGAGCTCCAAGATTTGCGAGGAGGTGGAGAGAATAGCACGGGACAGAGAGTACGATGG |

**Table S5: List of gene sequences used in the construction of Xpk/Pta pathway in *Y. lipolytica* strain YB-392. CO – Codon optimization; Xpk – Phosphoketolase; Pta - Phosphotransacetylase**

| **Gene name** | **Sequence** |
| --- | --- |
| Bs*PTA*(v1) (CO to *S. cerevisiae* using GeneArt) | ATGGCCGATTTGTTCTCTACCGTTCAAGAAAAAGTTGCTGGTAAGGATGTCAAGATCGTTTTTCCAGAAGGTTTGGACGAAAGAATTTTGGAAGCTGTTTCTAAATTGGCCGGTAACAAGGTTTTGAACCCAATCGTTATTGGTAACGAAAACGAAATTCAAGCCAAGGCCAAAGAATTGAACTTGACTTTGGGTGGTGTTAAGATCTACGATCCACATACTTATGAAGGTATGGAAGATTTGGTTCAAGCCTTCGTTGAAAGAAGAAAAGGTAAGGCTACTGAAGAACAAGCTAGAAAAGCTTTGTTAGACGAAAACTACTTCGGTACTATGTTGGTCTACAAAGGTTTGGCTGATGGTTTGGTTTCTGGTGCTGCTCATTCTACTGCTGATACTGTTAGACCAGCATTGCAAATCATCAAGACAAAAGAAGGTGTCAAAAAGACCTCCGGTGTTTTCATTATGGCTAGAGGTGAAGAACAATACGTTTTCGCTGATTGCGCTATTAACATTGCTCCAGATTCTCAAGATTTGGCCGAAATTGCTATTGAATCTGCTAACACTGCTAAGATGTTCGACATTGAACCTAGAGTTGCTATGTTGTCATTCTCTACAAAAGGTTCTGCTAAGTCTGACGAAACTGAAAAGGTTGCTGATGCAGTTAAGATCGCTAAAGAAAAAGCTCCAGAATTGACCTTGGATGGTGAATTTCAATTTGATGCTGCTTTCGTTCCATCCGTTGCTGAAAAAAAAGCACCAGATTCTGAAATCAAGGGTGATGCCAATGTTTTCGTATTCCCATCTTTAGAAGCTGGTAACATCGGTTACAAGATTGCTCAAAGATTGGGTAACTTTGAAGCTGTTGGTCCAATATTGCAAGGTTTGAATATGCCAGTTAACGATTTGTCTAGAGGTTGCAATGCAGAAGATGTTTACAACTTGGCTTTGATTACTGCTGCTCAAGCTTTGTAA |
| Ts*PTA*(v1) (CO to *S. cerevisiae* using GeneArt) | ATGTCCATCATCCAAAACATCATCGAAAAGGCCAAGTCCGATAAGAAGAAAATCGTTTTGCCAGAAGGTGCTGAACCTAGAACTTTGAAAGCTGCTGAAATCGTCTTGAAAGAAGGTATTGCTGATTTGGTCTTGTTGGGTAACGAAGACGAAATTAGAAATGCTGCCAAGGATTTGGATATCTCCAAGGCCGAAATTATCGATCCAGTTAAGTCTGAAATGTTCGACAGATACGCTAACGACTTCTACGAATTGAGAAAGAACAAGGGTATCACCTTGGAAAAGGCTAGAGAAACCATTAAGGACAACATCTACTTCGGTTGCATGATGGTCAAAGAAGGTTACGCTGACGGTTTGGTTTCTGGTGCTATTCATGCTACAGCTGATTTGTTAAGACCAGCCTTCCAAATTATCAAAACTGCTCCAGGTGCTAAGATCGTCAGTTCATTTTTCATTATGGAAGTCCCAAACTGCGAATACGGTGAAAATGGTGTTTTTTTGTTCGCTGATTGTGCCGTTAATCCATCTCCAAATGCTGAAGAATTGGCTTCCATTGCTGTTCAATCTGCTAATACTGCTAAGAATTTGTTGGGTTTCGAACCTAAGGTTGCCATGTTGTCTTTTTCAACAAAAGGTTCCGCTTCCCATGAATTGGTTGATAAGGTTAGAAAGGCTACCGAAATCGCCAAAGAATTGATGCCAGATGTTGCTATTGATGGTGAATTACAATTGGATGCTGCCTTGGTAAAAGAAGTTGCTGAATTGAAAGCTCCAGGTTCAAAAGTTGCTGGTTGTGCTAATGTTTTGATCTTCCCAGACTTACAAGCTGGTAACATTGGTTACAAGTTGGTTCAAAGATTGGCTAAGGCTAATGCCATTGGTCCAATTACTCAAGGTATGGGTGCTCCAGTTAATGATTTGTCTAGAGGTTGTTCCTACAGAGATATCGTTGATGTTATTGCTACTACCGCTGTTCAAGCTCAATAA |
| Ts*PTA*(v2) (CO to *Y. lipolytica* using GeneArt) | ATGTCCATCATCCAGAACATCATCGAGAAGGCCAAGTCTGACAAGAAGAAGATCGTTCTGCCCGAGGGCGCTGAGCCCCGAACTCTGAAGGCCGCCGAGATCGTGCTGAAGGAAGGCATTGCCGACCTGGTGCTGCTGGGCAACGAGGACGAGATCCGAAACGCCGCCAAGGACCTGGACATCTCTAAGGCCGAGATCATCGACCCCGTGAAGTCTGAGATGTTCGACCGATACGCCAACGACTTCTACGAGCTGCGAAAGAACAAGGGCATCACCCTGGAAAAGGCCCGAGAGACTATCAAGGACAACATCTACTTCGGCTGCATGATGGTCAAGGAAGGCTACGCCGACGGCCTGGTGTCTGGCGCCATCCACGCCACCGCCGACCTGCTGCGACCCGCCTTCCAGATCATCAAGACTGCCCCTGGCGCCAAGATCGTGTCCTCGTTCTTCATCATGGAAGTGCCCAACTGCGAGTACGGCGAGAACGGCGTGTTCCTGTTCGCCGACTGCGCTGTGAACCCCTCGCCTAACGCCGAGGAACTGGCCTCTATCGCCGTGCAGTCTGCCAACACCGCTAAGAACCTGCTGGGCTTCGAGCCCAAGGTGGCCATGCTGTCTTTCTCGACCAAGGGCTCTGCCTCTCACGAGCTGGTGGACAAGGTGCGAAAGGCTACCGAGATCGCCAAGGAACTGATGCCCGACGTGGCCATCGACGGCGAACTGCAGCTGGACGCCGCTCTGGTGAAGGAAGTGGCCGAGCTGAAGGCTCCCGGCTCTAAGGTGGCCGGCTGCGCCAACGTGCTGATCTTCCCCGACCTGCAGGCCGGCAACATCGGCTACAAGCTGGTGCAGCGACTGGCCAAGGCCAACGCCATCGGACCCATCACTCAAGGCATGGGCGCTCCCGTGAACGACCTGTCTCGAGGCTGCTCTTACCGAGACATCGTGGACGTGATCGCCACCACCGCTGTGCAGGCCCAGTAA |
| Ca*XPK*(v1) (CO to *Y. lipolytica* using ATGme) | ATGCAGTCCATCATCGGTAAACACAAGGATGAAGGTAAGATTACCCCTGAATACTTGAAGAAGATTGACGCTTATTGGCGAGCTGCCAACTTTATTTCTGTCGGTCAGCTTTACTTGTTGGACAACCCTTTGTTGCGAGAACCTTTGAAACCTGAACACTTGAAGCGAAAGGTTGTTGGTCACTGGGGTACTATTCCTGGTCAGAACTTCATCTACGCCCACTTGAACCGAGTCATCAAAAAGTACGATTTGGACATGATCTACGTTTCTGGTCCTGGTCACGGTGGTCAGGTTATGGTTTCTAACTCTTACTTGGACGGTACTTACTCCGAAGTTTACCCTAACGTTTCCCGAGATTTGAACGGTTTGAAGAAGTTGTGCAAGCAGTTTTCATTCCCTGGTGGTATCTCTTCACACATGGCTCCTGAAACTCCTGGTTCTATTAACGAAGGTGGTGAATTGGGTTATTCCTTGGCTCACTCTTTTGGTGCTGTTTTCGATAACCCTGATTTGATTACTGCTTGCGTTGTTGGTGATGGTGAAGCTGAAACTGGTCCTTTGGCTACATCTTGGCAGGCTAACAAATTTTTGAACCCTGTTACTGATGGTGCCGTTTTGCCTATTCTTCACTTGAACGGTTACAAGATCTCCAACCCTACTGTCTTGTCTCGAATTCCTAAGGACGAATTGGAAAAGTTCTTCGAAGGTAACGGTTGGAAGCCTTACTTTGTTGAAGGTGAAGATCCTGAAACCATGCACAAGTTGATGGCTGAAACTTTGGATATCGTCACCGAAGAAATCTTGAACATTCAGAAGAACGCCCGAGAAAACAACGATTGCTCTCGACCTAAATGGCCTATGATCGTTTTGCGAACTCCTAAAGGTTGGACTGGTCCTAAATTCGTTGATGGTGTTCCTAACGAAGGTTCTTTTCGAGCACACCAGGTTCCTTTGGCAGTTGATCGATACCACACCGAAAACTTGGACCAGTTGGAAGAATGGTTGAAGTCTTACAAGCCTGAAGAACTTTTCGACGAAAACTACCGATTGATCCCTGAACTTGAAGAATTGACCCCTAAGGGTAACAAGCGAATGGCTGCTAACTTGCACGCTAACGGTGGTTTGTTGCTTCGAGAATTGCGAACCCCTGATTTCCGAGATTACGCTGTTGATGTTCCTACACCTGGTTCAACTGTTAAGCAGGATATGATCGAATTGGGTAAATACGTCCGAGATGTCGTCAAGTTGAACGAAGATACACGAAACTTCCGAATCTTCGGTCCTGACGAAACTATGTCTAACCGATTGTGGGCTGTCTTTGAAGGTACTAAGCGACAGTGGTTGTCCGAAATCAAAGAACCTAACGACGAATTCTTGTCCAACGATGGTCGAATCGTTGACTCTATGTTGTCTGAACACTTGTGTGAAGGTTGGCTTGAAGGTTACTTGTTGACTGGTCGACACGGTTTTTTTGCCTCTTACGAAGCTTTCTTGCGAATCGTCGATTCCATGATTACCCAGCACGGTAAATGGTTGAAAGTCACCTCTCAGTTGCCTTGGCGAAAGGATATTGCTTCCTTGAACTTGATTGCCACCTCTAACGTTTGGCAGCAGGATCACAACGGTTATACCCACCAGGACCCTGGTTTGTTGGGTCACATAGTTGATAAGAAGCCTGAAATCGTTCGAGCTTATTTGCCTGCTGATGCTAACACTTTGTTGGCCGTTTTTGATAAGTGCTTGCACACCAAGCACAAGATCAACTTGTTGGTTACCTCTAAACACCCTCGACAGCAGTGGCTTACTATGGATCAGGCCGTTAAGCACGTTGAACAGGGTATTTCTATTTGGGATTGGGCTTCTAACGATAAGGGTCAGGAACCTGATGTTGTTATTGCTTCCTGTGGTGATACTCCTACTTTGGAAGCTTTGGCTGCTGTTACCATTCTTCACGAACACTTGCCTGAATTGAAGGTCCGATTCGTTAACGTTGTTGACATGATGAAGTTGTTGCCTGAAAACGAACACCCTCACGGTTTGTCTGATAAGGATTACAACGCTTTGTTCACTACCGATAAGCCTGTTATTTTTGCCTTTCACGGTTTCGCCCACTTGATCAACCAGTTGACCTACCACCGAGAAAACCGAAACTTGCACGTTCACGGTTACATGGAAGAAGGTACAATTACTACTCCTTTCGACATGCGAGTCCAGAACAAGTTGGACCGATTCAACTTGGTTAAGGACGTCGTTGAAAACTTGCCTCAGCTTGGTAACCGAGGTGCCCACTTGGTTCAGTTGATGAACGATAAGTTGGTCGAACACAACCAGTATATCCGAGAAGTTGGTGAAGATTTGCCTGAAATTACCAACTGGCAGTGGCACGTTTGA |
| Ca*XPK*(v2) (manually CO to *Y. lipolytica* by replacing all possible codons at frequency ≤2% using ATGme) | ATGCAGTCCATCATCGGAAAGCACAAGGATGAGGGAAAGATTACCCCCGAGTACCTCAAGAAGATTGACGCTTACTGGCGAGCTGCCAACTTCATTTCTGTCGGACAGCTCTACCTCCTCGACAACCCCCTCCTCCGAGAGCCCCTCAAGCCCGAGCACCTCAAGCGAAAGGTGGTGGGACACTGGGGAACCATTCCCGGACAGAACTTCATCTACGCCCACCTCAACCGAGTCATCAAGAAGTACGATCTCGACATGATCTACGTGTCTGGACCCGGACACGGAGGACAGGTGATGGTGTCTAACTCTTACCTCGACGGAACCTACTCCGAGGTGTACCCCAACGTGTCCCGAGATCTCAACGGACTCAAGAAGCTCTGCAAGCAGTTCTCTTTCCCCGGAGGAATCTCTTCTCACATGGCTCCCGAGACCCCCGGATCTATTAACGAGGGAGGAGAGCTCGGATACTCCCTCGCTCACTCTTTCGGAGCTGTGTTCGATAACCCCGATCTCATTACCGCTTGCGTGGTGGGAGATGGAGAGGCTGAGACCGGACCCCTCGCTACCTCTTGGCAGGCTAACAAGTTCCTCAACCCCGTGACCGATGGAGCCGTGCTCCCCATTCTCCACCTCAACGGATACAAGATCTCCAACCCCACCGTCCTCTCTCGAATTCCCAAGGACGAGCTCGAGAAGTTCTTCGAGGGAAACGGATGGAAGCCCTACTTCGTGGAGGGAGAGGATCCCGAGACCATGCACAAGCTCATGGCTGAGACCCTCGATATCGTCACCGAGGAGATCCTCAACATTCAGAAGAACGCCCGAGAGAACAACGATTGCTCTCGACCCAAGTGGCCCATGATCGTGCTCCGAACCCCCAAGGGATGGACCGGACCCAAGTTCGTGGATGGAGTGCCCAACGAGGGATCTTTCCGAGCTCACCAGGTGCCCCTCGCTGTGGATCGATACCACACCGAGAACCTCGACCAGCTCGAGGAGTGGCTCAAGTCTTACAAGCCCGAGGAGCTCTTCGACGAGAACTACCGACTCATCCCCGAGCTCGAGGAGCTCACCCCCAAGGGAAACAAGCGAATGGCTGCTAACCTCCACGCTAACGGAGGACTCCTCCTCCGAGAGCTCCGAACCCCCGATTTCCGAGATTACGCTGTGGATGTGCCCACCCCCGGATCTACCGTGAAGCAGGATATGATCGAGCTCGGAAAGTACGTCCGAGATGTCGTCAAGCTCAACGAGGATACCCGAAACTTCCGAATCTTCGGACCCGACGAGACCATGTCTAACCGACTCTGGGCTGTCTTCGAGGGAACCAAGCGACAGTGGCTCTCCGAGATCAAGGAGCCCAACGACGAGTTCCTCTCCAACGATGGACGAATCGTGGACTCTATGCTCTCTGAGCACCTCTGTGAGGGATGGCTCGAGGGATACCTCCTCACCGGACGACACGGATTCTTCGCCTCTTACGAGGCTTTCCTCCGAATCGTCGATTCCATGATTACCCAGCACGGAAAGTGGCTCAAGGTCACCTCTCAGCTCCCCTGGCGAAAGGATATTGCTTCCCTCAACCTCATTGCCACCTCTAACGTGTGGCAGCAGGATCACAACGGATACACCCACCAGGACCCCGGACTCCTCGGACACATTGTGGATAAGAAGCCCGAGATCGTGCGAGCTTACCTCCCCGCTGATGCTAACACCCTCCTCGCCGTGTTCGATAAGTGCCTCCACACCAAGCACAAGATCAACCTCCTCGTGACCTCTAAGCACCCCCGACAGCAGTGGCTCACCATGGATCAGGCCGTGAAGCACGTGGAGCAGGGAATTTCTATTTGGGATTGGGCTTCTAACGATAAGGGACAGGAGCCCGATGTGGTGATTGCTTCCTGTGGAGATACCCCCACCCTCGAGGCTCTCGCTGCTGTGACCATTCTCCACGAGCACCTCCCCGAGCTCAAGGTCCGATTCGTGAACGTGGTGGACATGATGAAGCTCCTCCCCGAGAACGAGCACCCCCACGGACTCTCTGATAAGGATTACAACGCTCTCTTCACCACCGATAAGCCCGTGATTTTCGCCTTCCACGGATTCGCCCACCTCATCAACCAGCTCACCTACCACCGAGAGAACCGAAACCTCCACGTGCACGGATACATGGAGGAGGGAACCATTACCACCCCCTTCGACATGCGAGTCCAGAACAAGCTCGACCGATTCAACCTCGTGAAGGACGTCGTGGAGAACCTCCCCCAGCTCGGAAACCGAGGAGCCCACCTCGTGCAGCTCATGAACGATAAGCTCGTCGAGCACAACCAGTACATCCGAGAGGTGGGAGAGGATCTCCCCGAGATTACCAACTGGCAGTGGCACGTGTGA |
